# A Negative Reputation Reduces Trust Despite Trustworthy Behavior

**DOI:** 10.1111/psyp.70102

**Published:** 2025-07-01

**Authors:** Kilian Stenzel, Martin Weiß, Grit Hein

**Affiliations:** ^1^ Division of Clinical Psychology and Psychotherapy, Department of Psychology Philipps‐University of Marburg Marburg Germany; ^2^ Center of Mental Health, Department of Psychiatry, Psychosomatic and Psychotherapy, Translational Social Neuroscience Unit University of Würzburg Würzburg Germany; ^3^ Department of Psychology I: Clinical Psychology and Psychotherapy Institute of Psychology, University of Würzburg Würzburg Germany

**Keywords:** EEG, reputation, social decision making, theta, trust

## Abstract

Interpersonal trust decisions are guided by reputation. However, it remains unclear how positive and negative prior reputations that are inconsistent with a partner's behavior are integrated at the behavioral and neural levels and how this informs daily trust decisions. In this two‐part study, 54 subjects first played an iterated 20‐trial Trust Game with four anonymous partners introduced as “cooperative” or “individualistic” while EEG was recorded. The partners’ behavior then either confirmed or contradicted this prior reputation. Subsequently, the subjects completed a three‐day ecological assessment measuring trust in daily interactions. According to the results, negative prior reputations were associated with fewer trust decisions, even after being contradicted by cooperative behavior. The frequency of trust decisions remained high if positive prior reputations were confirmed and decreased if they were contradicted. Trial‐by‐trial analyses showed that negative priors were still related to a lower trust choice probability, even if they were contradicted in the previous trial, paralleled by a decrease in fronto‐lateral theta. Mean trust levels across laboratory conditions were descriptively associated with mean trust levels in daily interactions. In sum, these findings indicate that a person with a negative prior reputation is less trusted, even if this person behaves in a positive way.

## Impact Statement

Our study is the first to demonstrate that negative prior reputations not only decrease trust likelihood despite contradictory evidence but also correlate with reduced fronto‐lateral theta activity. Thereby, it highlights the resistance to revising trust when reputational bias is negative. It is also the first to report data on the prediction of daily trust decisions by theta power differences during laboratory trust games.

## 1 Introduction

Interpersonal trust is crucial for dyadic, group, and societal cooperation (Krueger and Meyer‐Lindenberg 2019). It is defined as the “expectation of other's benevolent motives” (Balliet and Van Lange 2013, p. 1091) and requires the acceptance of risk in uncertain situations (Mayer et al. 1995), transforming the fear of betrayal into positive expectations of reciprocity (Declerck et al. 2013). Trust involves three perspectives: the trustor's general willingness to trust, the trustee as perceived by the trustor, notably influenced by their reputation (Delgado et al. 2005; Fouragnan et al. 2013), and the dynamics of the trustor‐trustee relationship, influenced by interaction, closeness, and social interdependence (Weiss et al. 2021). In laboratory research, trust is standardly operationalized by the Trust Game (TG; Berg et al. 1995), in which a trustor is initially endowed with a certain number of points. The trustor then decides whether to keep the points or to share them with an interaction partner (trustee), who previously received the same number of points. In the first case, the trustor keeps the points. In the latter case, the points are tripled, and the trustee decides whether to send half of them back or keep all. Hence, trust is operationally defined as the number of choices in which the trustor sends the money to the trustee. The iterative version of the TG is sensitive to the dynamics of the trust formation process (Chang et al. 2010). The disposition to trust is related to general trust, belief in human benevolence, and social value orientation (SVO), which influence prosocial behavior (Murphy et al. 2011; Yamagishi and Yamagishi 1994). Higher levels of SVO typically lead to higher expectations of others’ benevolent motives and prosocial behavior in situations of social interdependence such as the TG (Pletzer et al. 2018).

In the absence of previous interactions, trustors rely on social signals and information about a trustee's “reputation” to guide trust decisions (Delgado et al. 2005; Fouragnan et al. 2013). Thus, reputation is exceedingly important to daily interactions and transactions with strangers. Trustee reputation has previously been induced by symbols resembling high or low SVO scores (Fouragnan et al. 2013) or by past TG return rates (Rezlescu et al. 2012). Reputational priors—information about a trustee's reputation available before an interaction—guide initial trust decisions while increasing medial prefrontal cortex activity, with positive information leading to greater trust (Fouragnan et al. 2013). Hence, there are neurophysiological processes that may predict how prior reputation shapes trust decisions. It was also found that learning is facilitated when feedback is consistent with the reputational prior and previous social interactions (either negatively consistent or positively consistent; Fareri et al. 2012). However, these studies only investigated the effect of prior reputation on trust decisions and its neural precursors, often neglecting whether and how these effects are altered by behavior that is consistent vs. inconsistent with reputation. This, however, would be important to gain a deeper understanding of everyday trust decisions, which are made by integrating contextual information, such as reputation. Hence, the current study seeks to address this shortcoming by investigating how electrocortical correlates may differentially inform trust behavior between these conditions. The behavioral dynamics of trust and the cognitive processing of reputational priors can be effectively examined using electroencephalography (EEG) in combination with standardized laboratory tasks, such as the TG. The candidate neural systems underlying these processes—specifically the cognitive control and reward systems—have already successfully been studied using these means (e.g., Arabadzhiyska et al. 2021; Billeke et al. 2013; Hu et al. 2018).

First, it has been found that beta power likely reflects a cognitive inhibition mechanism (Aron 2011; Huster et al. 2013) activated if decision preferences must be maintained when prior stimulus information conflicts with the current optimal behavioral strategy (Engel and Fries 2010). It could be shown that increased beta power was more pronounced in persons with high trait social risk‐seeking after distrust choices compared to trust choices in the TG (Wang et al. 2017). The authors suggested that this group must exert higher cognitive control to make a distrusting choice and deviate from their impulsive behavioral strategy. More recent studies found that higher fronto‐central beta (FCB) power during the decision generation phase of a TG predicted a higher likelihood of a trust choice during the decision output (Fu et al. 2019; Wang et al. 2021). Unfortunately, these studies did not vary the reciprocity rate of the trustee, fixing it to 50%, nor did they introduce inconsistent prior information. This manipulation, however, would be needed to generalize the effect.

Second, midfrontal theta (MFT) power serves as an indicator of trust‐related cognitive control mechanisms (Huster et al. 2013) signaling the need for control under uncertainty (Cavanagh et al. 2010; Cavanagh and Frank 2014; Cavanagh and Shackman 2015). MFT facilitates the linkage between prediction error signals and behavioral adaptation in the *reward system* (Cavanagh et al. 2010). A recent study found that MFT was higher following loss than gain outcome evaluations in the TG, whereby the MFT difference was attenuated following trust compared to distrust choices (Hu et al. 2018), suggesting that MFT‐related processes are more sensitive to negative expectation violations in the sense of a loss aversion. This suggests that MFT reflects a mechanism that ensures flexible performance adjustments by adapting to feedback and conflicts (Ridderinkhof et al. 2004) that may be translated to trust situations. Processes related to *fronto‐lateral* theta (FLT) power are also associated with maintaining control amidst conflicts between stimuli and behavioral regimes (Krämer et al. 2013). Furthermore, FLT was associated with RT speeding in response to correct choices instead of slowing down in response to erroneous choices (Cavanagh et al. 2010), which might suggest that FLT processes are more sensitive to positive expectation violations. These suggested theta processes are highly relevant to interpersonal trust if reputation contradicts actual behavior, although they have rarely been investigated to date. Hence, this study seeks to investigate whether and how the above‐described findings concerning the role of theta translate to the social domain by adopting a TG paradigm.

The summarized studies were all conducted in the laboratory. Therefore, it is unclear whether and how the observed effects translate to trust decisions in real life. Previous studies (Glaeser et al. 2000; Weiss et al. 2021) were unable to use laboratory trust rates to predict trust decisions in everyday life TGs. However, both studies used different TGs for the laboratory task than for the daily life task. Hence, a thorough comparison of two similar TG outcomes is missing. Also, it still remains unclear whether laboratory TGs can inform ecological TGs and how. Hence, this study also seeks to shed light on this question. Recent advances in ecological momentary assessment (EMA) methods provide new possibilities to investigate the relationship between neurophysiological changes during laboratory tasks and everyday social experiences and behavior: Dell'Acqua et al. (2024) found that the reward positivity in response to social rewards in the laboratory moderated the link between positive events of social support and higher positive affect in everyday life. Higher reward positivity to a monetary laboratory task was also associated with more daily positive affect (Duttweiler et al. 2024). Hence, combining data from established EEG markers with EMA can provide novel insights into the relationship between the disposition to trust, the integration of inconsistent information, and daily trust decisions across social contexts. Research in this area is still scarce, especially for processes underlying beta and theta power.

Taking previous evidence together, it remains unclear how trust decisions and the underlying neurophysiological processes are shaped by positive and negative reputations and reputation‐consistent or inconsistent behavior, and whether laboratory TG behavior and associated neurophysiological activity can inform daily trust decisions. To shed light on these questions, the first part of our research used a modified version of the TG combined with EEG in the laboratory. In the second part of our study, the same participants took EMAs, enabling the investigation of trust decisions in daily life.

Based on previous evidence showing that trust is influenced by reputation (Delgado et al. 2005; Fouragnan et al. 2013) and the dynamics of the trustor‐trustee relationship (Weiss et al. 2021), we assumed that the frequency of trust decisions is modulated by the interaction between the trustors' reputations (i.e., cooperative or individualistic) and their actual behavior (cooperative or individualistic, consistent or inconsistent with the prior).

This study aimed to replicate that higher beta power predicts higher trust choice likelihood across varying consistency and valence of prior reputational information in hypothesis 1 (H1, Fu et al. 2019; Wang et al. 2021). We predicted that prior‐inconsistent individualistic behavior (worse than expected vs. bad as expected) is related to a decreased likelihood of making a trust choice, accompanied by higher MFT during TG feedback (Hu et al. 2018) in hypothesis 2 (H2). In contrast, we predicted that prior‐inconsistent cooperative behavior (better than expected vs. good as expected) is related to an increased likelihood of a trust choice, paralleled by higher fronto‐lateral theta power (FLT) during TG feedback (Cavanagh et al. 2010; Krämer et al. 2013) in hypothesis 3 (H3). Regarding the link between the laboratory and EMA data, we hypothesized that the trust choice frequency in real‐life interactions would increase with the frequency of trust choices in a laboratory TG in hypothesis 4 (H4). As beta power indicates the maintenance of behavioral strategies, social risk‐seeking, and trust‐promoting behavior (Aron 2011; Engel and Fries 2010; Huster et al. 2013; Wang et al. 2017), we assumed that increased FC beta during the decision generation of a laboratory‐based TG predicts higher daily trust levels in hypothesis 5 (H5). Finally, inspired by converging findings linking theta power to flexible behavioral adjustment in situations of conflicting information (Cohen and Donner 2013; Nigbur et al. 2012; Ridderinkhof et al. 2004), we investigated whether increased theta power differences (MFT and FLT, resp.) between feedback of expectation‐consistent (good as expected and bad as expected, resp.) and expectation‐inconsistent (better than expected and worse than expected, resp.) partners' behavior predict higher daily trust levels in hypothesis 6 (H6).

## 2 Methods

### 2.1 Sample, Data Exclusion, and Pre‐Registration

Owing to the novelty of our design, no comparable studies were found to precisely estimate the power for the translational effect predicting everyday trust decisions by theta power differences. As we expected this to be the smallest effect, we based our sample size estimation on this effect. Earlier studies on the relationship between empathy‐related neural activation (in fMRI) and daily prosocial choices suggest effect sizes between 0.38 ≤ *r* ≤ 0.45 (Morelli et al. 2014; Rameson et al. 2012; Vekaria et al. 2020) with an average of *r* = 0.42 across studies. A power analysis using the R function “pwr.r.test” (Champely 2020) based on a power of 0.90 and a two‐tailed *α* of 0.05 indicated that 55 participants would be needed to detect an simple linear regression effect of *r* = 0.42. This is even more conservative compared to a recent review on studies linking fMRI with EMA, where the average sample size across 71 studies was 51 (Gadassi‐Polack et al. 2024). This effect size estimate is also more conservative than the effect size expected for using MFT changes to negative feedback to predict behavioral adaptation: Meta‐analytic evidence (Cavanagh and Shackman 2015) suggests a mean effect size of *r* = 0.69. To account for outliers and to balance our sample, a total of 60 participants completed the study. One participant met the exclusion criterion for clinical depression and was thus excluded from participation before the EMA period. The EEG datasets of five participants could not be processed because of bad data and were thus exempt from further analysis. Another dataset did not meet the preregistered threshold of at least ten completed EMA prompts. Hence, the sample to answer the hypotheses involving laboratory variables consisted of 55 participants and the sample to answer the combined hypotheses consisted of 54 participants. Individuals who could not operate a smartphone, had no German language proficiency, had a self‐reported clinical diagnosis of social anxiety disorder, had a self‐reported clinical diagnosis of depression, had a depression screening value of > 20 (using the “Allgemeine Depressions‐Skala – Kurzform”, Lehr et al. 2008), or had a self‐reported clinical diagnosis of schizophrenia were not allowed to participate. The depression screening value threshold was erroneously preregistered as ≥ 16, although the literature (Lehr et al. 2008) recommends ≥ 18. However, this threshold did not serve a critical purpose and was introduced to make sure participants would not lack motivation to participate in the EMA survey. Hence, we finally applied a more liberal threshold of > 20, and asked participants if they felt able to take the surveys. As preregistered, a TG trial was discarded if its respective RT was shorter than 0.2 s or exceeded 1.5 s. It was also preregistered that if more than 30% of trials of a single block must be discarded, its data would not be used to answer hypotheses H1 through H5. And finally, if more than 20% of trials of the entire TG would have to be discarded, the participant would be excluded from further analysis and participation in the EMA part. However, lacking a pilot study these percentual cut‐offs could not be met by any of the subjects and were thus not applied.

Participants provided written informed consent. For the on‐site experimental part, subjects received a fixed financial compensation of 15 Euro (or course credit). In addition, 1–3 Euro could be gained depending on the TG result. To this end, the point average across trials was taken with 10 points referring to 1 cent. However, as the return rate of the trustee was unpredictable, 1.5 Euro would realistically be gained. For the EMA part, 0.5 Euro per prompt could be gained. Hence, in sum, the payoff ranged between 16.5 and 28.5 Euro. If course credit was obtained, the financial compensation ranged between 1.5 Euro and 13.5 Euro. Prior to data collection, the study was preregistered on the Open Science Framework (Stenzel and Weiß 2023). However, not all results are presented in the main text or the supporting information (SI) —S1: as the current study focuses on psychophysiological correlates, the results concerning the preregistered hypotheses which only use EMA data (*preregistered as* H1.6, H1.7) are neither presented in the main text nor the SI. Furthermore, as the current study focuses particularly on frequency bands to predict trust decisions in the laboratory and daily interactions, the results concerning the preregistered hypotheses, which only use event‐related potentials (*preregistered as* H1.4, H1.5) and response time data (*preregistered* as H1.2a, H1.3a) as outcome variables are presented in the SI (Tables S12–S15).

### 2.2 Task and Procedure

On arrival, the participants were introduced to the experimental procedure. Next, they were informed about the TG, conducted ten practice trials, and answered the SVO (Fouragnan et al. 2013; Murphy et al. 2011; Van Lange 1999, c.f. Figure S2). The experiment was divided into five blocks, each with a different anonymous partner. Unknown to the participants, the decisions of their partners were predefined. Each block consisted of an “induction phase” of the reputational prior (cooperative/neutral/individualistic). In this phase, the participants saw their own and the trustee's SVO scores (for details, see SI). The cooperative reputational prior refers to a jittered SVO score of 89 ± 2 (unusually high), the neutral prior to an SVO score of 76 (average), and the individualistic prior to a jittered SVO score of 63 ± 2 (unusually low).

In each block, participants completed 20 trials of an iterated TG with varying cooperation rates for the trustee (cooperative: 80%; neutral: 50%; individualistic: 20%). These cooperation rates were either consistent or inconsistent with the reputational prior, resulting in five blocks: cooperative‐consistent (CC), cooperative‐inconsistent (CI), individualistic‐consistent (IC), individualistic‐inconsistent (II), and neutral‐consistent (NC). The order of the blocks was pseudo‐counterbalanced, such that each block appeared at each position at least once (see Table S1). However, the NC block was always placed in the third position to minimize learning in the cross‐over design and did not serve as a control condition. It reflects stable interindividual differences that influence trust decisions similarly across blocks and conditions, and thus are unlikely to contribute to the differential effects we tested in our main hypotheses. Given that we had no a priori hypotheses regarding the effects of the neutral block, we refrained from including the data in our analyses.

**FIGURE 1:**
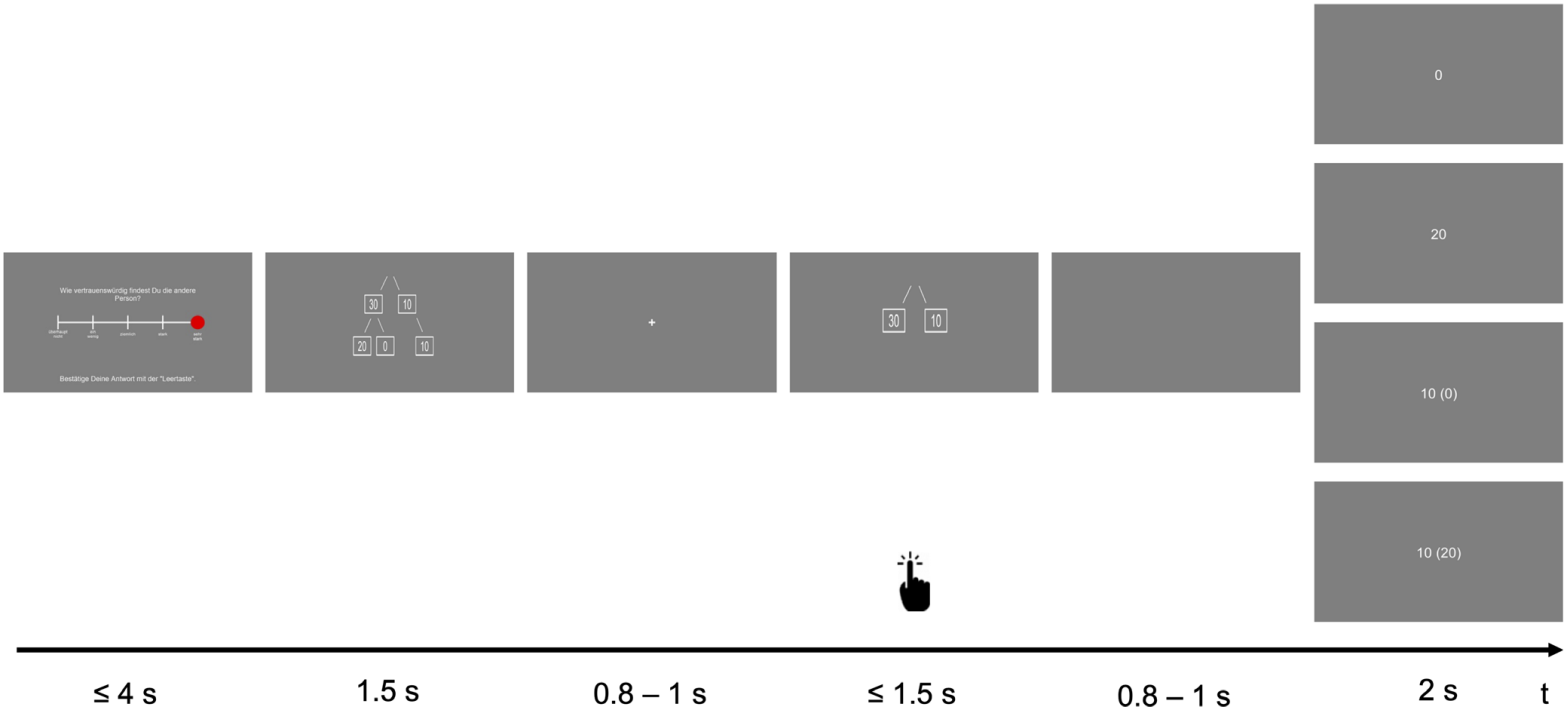
Exemplary trial of the Trust Game. First, participants saw a five‐point rating referring to the trustworthiness of their anonymous interaction partner. Second, they were shown a tree structure depicting all potential outcomes of the Trust Game (0, 10, 20). Third, they saw a fixation cross. Forth, the choice options were shown, and the trust or distrust choice was made. Lastly, after a blank slide, participants received feedback of their outcome which depended on their previous choice. In case of a previous distrust choice, the number in brackets indicates the hypothetical payoff if they would have made a trust choice instead.

Each TG trial started with a rating of the trustee's trustworthiness asking to indicate “How trustworthy do you consider this person?” on a five‐point Likert scale (see Figure 1; Figure S3), which was later used as a manipulation check. After confirming the rating with the space bar or a maximum of 4 s, a decision tree was displayed for 1.5 s revealing the payoff‐structure of the TG (“decision generation stage”). After a jittered inter‐stimulus interval (ISI) with fixation cross of 0.8–1 s, the choice options were displayed. In each round, the participant was endowed with 10 points (translating to 1 cent). Then, the participant could choose whether to keep or send the points (“choice execution stage”) using the “left” or “right” arrow keys within a 1.5 s interval (orientation randomized across participants). In the case of a trust choice, the points were tripled, and the trustee decided whether to send half of it back (i.e., both get 20) or keep all (i.e., trustor gets 0, trustee gets 40). After a blank jittered ISI of 0.8–1 s, the feedback appeared for 2 s which could either be “0”, “20”, “10 (0)” or “10 (20)”. Numbers in brackets indicated the payoff if the participant would have chosen to trust. This made sure, that participants learned about the trustees’ choices even when chose not to trust and enabled us to keep the number of trials fixed (Fouragnan et al. 2013).

We used the following labeling to facilitate the interpretation of the results: if the partner's reputational prior and actual behavior were both cooperative, the trial was classified as “good as expected”. If the partner's reputational prior and the actual behavior were both individualistic, the trial was classified as “bad as expected”. If the partner's reputational prior was cooperative but the actual behavior of the trial was individualistic, the trial was classified as “worse than expected”. If the partner's reputational prior was individualistic but the actual behavior of the trial was cooperative, the trial was classified as “better than expected”. In total, participants performed 100 trials of the TG (i.e., 20 trials in five blocks).

After the experiment, participants answered additional questionnaires (see “2.4 Questionnaires”), were equipped with study smartphones, and were instructed for the EMA period, which lasted for three consecutive days (starting the day after the on‐site experimental session). There were eight prompts per day within a 14‐h interval appearing at random times with an inter‐prompt interval of at least 45 min. The start time of the 14‐h interval was determined by the participant's wake‐up time. The period started an hour after the wake‐up time but was no later than 10 am. Finally, two example scenarios were completed, and the smartphone was handed over. After the final day of the assessment, the participants returned their smartphones and received financial compensation.

### 2.3 Apparatus

The EEG (sampling rate: 1000 Hz) was recorded using BrainVision Recorder software (v1.24.0001, Brain Products GmbH, Gilching, Germany) from 32 scalp positions according to the 10–20 system using Ag/AgCl active electrodes (ActiCap 32‐system) and a BrainAmp DC amplifier (Brain Products GmbH, Gilching, Germany). Electrodes recorded were “Fp1”, “Fp2”, “F7”, “F3”, “Fz”, “F4”, “F8”, “FC5”, “FC1”, “FC2”, “FC6”, “T7”, “C3”, “Cz”, “C4”, “T8”, “TP9”, “CP5”, “CP1”, “CP2”, “CP6”, “TP10”, “P7”, “P3”, “Pz”, “P4”, “P8”, “O1”, “Oz”, “O2”, “PO9”, “PO10”, “FCz”. Participants were seated 70 cm away from a 33.5 × 60 cm LCD‐backlight screen (27‐in., physical resolution: 3840 × 2160 pixels; final resolution: 1920 × 1080 pixels) in an unlit EEG‐booth.

### 2.4 Questionnaires

The respective German version of the following questionnaires captured traits: Trust was assessed using the “Inclusive General Trust Scale” (IGTS; Jasielska et al. 2021; Yamagishi et al. 2015) to distinguish trust beliefs from preferences. Personality characteristics were assessed using a brief measure of the Ten‐Item Personality Inventory (TIPI‐G; Gosling et al. 2003; Muck et al. 2007) derived from the Big Five framework (Costa and McCrae 1992). Symptoms of depression were assessed using the 15‐item version of the Center for Epidemiologic Studies Depression Scale (German: “Allgemeine Depressions‐Skala – Kurzform” ADS‐K; Hautzinger et al. 2012; Hautzinger and Bailer 1993). Finally, symptoms of social anxiety were assessed using the Social Interaction Anxiety Scale in its respective German translation (SIAS; Heimberg et al. 1992; Stangier et al. 1999). EMA prompts were delivered using the movisensXS app (Movisens GmbH) on Android study‐smartphones. Each prompt, the first question asked whether a social interaction had taken place within the previous 45 min. In case there was no social interaction, a survey on the most recent activity was presented making sure that the EMA burden was the same irrespective of social interaction presence. In case there was a social interaction, the following variables were acquired (in order of appearance): subjective wellbeing, subjective stress, context, medium, observers, and pleasantness of interaction. Variables concerning the interaction partner were: gender, age (guessed), type of relationship, target initials or nickname (to adjust for repeated interactions with the same person), familiarity, closeness, similarity, trustworthiness. Then, a brief measure of the Ten‐Item Personality Inventory (TIPI‐G; Gosling et al. 2003; Muck et al. 2007) regarding the perceived personality of the interaction partner was administered. Lastly, the outcome variable collected during the EMA survey was the binary choice in an imaginary one‐shot TG. Hereby, the participants were first asked to recall the TG from the laboratory. They were then asked to decide whether they would trust the person they had last met in everyday life as if the TG would be played. The decision tree options were displayed as in the experiment (Figure 1, fourth panel from left). By design, no feedback could be provided.

### 2.5 EEG Preprocessing

For preprocessing, the steps of the EPOS pipeline (Rodrigues et al. 2021) were taken using MATLAB and EEGlab (Delorme and Makeig 2004). The ground electrode was located at Fpz, and the reference electrode at FCz. Raw data were filtered with a 1–40 Hz Butterworth bandpass filter after statistical channel selection using the *z*‐value‐based channel rejection for probability, kurtosis, and frequency range from 1 to 125 Hz, using the threshold of *z* > 3.29 (Tabachnick and Fidell 2013). A first independent component analysis (ICA; Makeig et al. 2004) was computed, and bad trials were rejected using IC‐based *z*‐value artifact detection for probability and kurtosis (*z* > 3.29; Tabachnick and Fidell 2013). After this segment rejection, a second ICA was performed using ADJUST (Mognon et al. 2011) and MARA (Winkler et al. 2011) to automatically detect and reject the artifact components. Current source density transformation was then applied using the script provided by Cohen (2014b). Data were segmented from −1.0 to 1.0 s from relevant markers and baseline corrected with a baseline from −0.2 to 0 s to the respective event onset.

All frequency bands were extracted using morlet wavelets (Cohen 2019). First, MFT frequency was extracted from 4 to 8 Hz. Theta peaks were detected with automated peak detection for the mean frequency in all trials being “bad as expected” and “good as expected” on the electrode FCz in the same time window as the N2 (200–400 ms). This process was reiterated for all trials being “worse than expected” and “better than expected”. The peak was found at 343 ms. Second, FLT frequency was extracted from 4 to 8 Hz. FLT peaks were detected with automated peak detection for the mean frequency in all trials being “bad as expected” and “good as expected” on the electrodes FC5 and FC6, respectively. Again, the same time window as the N2 (200–400 ms) was used. This process was reiterated for all trials being “worse than expected” and “better than expected”. The peak was found at 259 ms for FC5 and at 250 ms for FC6. Third, FCB frequency was extracted from 14 to 21 Hz. FCB peaks were detected with automated peak detection for the mean frequency response for all trials on the electrode FCz from 200 to 400 ms. The peak was found at 234 ms. The process of peak detection for the ERPs can be found in the supporting information—S1, as the respective results are also reported there.

### 2.6 Analyses

All statistical analyses were conducted using RStudio v4.3.0 (2023 The R Foundation for Statistical Computing). We captured the effects of blockwise manipulation and the trial‐by‐trial manipulation using different variables and analyses: analyses capturing the blockwise manipulation of the prior used inconsistent blocks (cooperative‐inconsistent/individualistic‐inconsistent) as the predictor variable and the average frequency of trust decisions across participants as the outcome variable. For this, we used simple linear models estimated by “lm” of the “stats”‐package. Analyses capturing trial‐by‐trial effects used feedback from the previous trial (bad as expected/better than expected/good as expected/worse than expected) and the respective electrocortical signal (MFT or FLT) as predictor variables, and the individual trust choice as a binary outcome variable. We modeled these variables with logistic mixed models using the “lme4”‐package (Bates et al. 2015) with “glmer” and “logit link”. Participants were included as random intercepts. Continuous predictor variables were within‐subject centered. *P*‐values were obtained using the Satterthwaite approximation of degrees of freedom (Luke 2017). Analyses capturing the translational effects from lab to EMA used the mean trust choice rate across the experiment or the electrocortical correlates (average score of MFT and FLT) as predictors and the mean trust choice rate across the EMA as the outcome variable. Again, simple linear models were used. As a manipulation check, we tested whether the initial trustworthiness ratings (please note that these were *not* the binary TG trust decisions) of conditions with a cooperative reputational prior were significantly higher than those with an individualistic reputational prior using a one‐sided paired *t*‐test.

Missing data, unequal sample sizes, the violation of the independence of errors, and the non‐normal distribution of the outcome variable (i.e., binary choices, response times) were expected. However, these do not pose problems to multilevel models and were thus not addressed (Tabachnick and Fidell 2013). Random intercept coefficients and random slope coefficients were assessed for normal distribution by visually inspecting the QQ‐plot and statistically by using the Shapiro–Wilk test (Shapiro et al. 1968) on the random effects models. Predictor variables were centered preemptively to avoid the problem of multicollinearity (Kreft and de Leeuw 1998). Homogeneity of variance was assessed visually by plotting the predicted outcome values against the residuals and statistically by using the Levene test on all predictor levels (Levene 1960) (R‐package “car”; Fox et al. 2013). All assumption checks were only performed where applicable to the data. If an assumption of normal distribution was not met, the variable was transformed, so that the assumption was met (logarithmic or square root transformation). For the models concerning H1 through H3, the subject variable was entered as a random intercept to account for the repeated‐measures design. Also, the trial number nested within blocks was entered as a random slope because trial‐by‐trial learning differed between conditions. However, respective terms were only kept in case of convergence. To address H1, a multilevel logistic model was run, where the outcome was the binary trust choice of the current trial. The predictor was the mean‐centered FCB during the decision generation stage of the current trial. The subject variable was entered as a random intercept, and the trial number nested in block number was entered as a random slope. The model converged. To address H2, a multilevel logistic regression was run, where the outcome was the binary trust choice of the current trial. The predictors were feedback (only defection trials: “worse‐than‐expected” vs. “as‐expected”) of the previous trial, MFT of the previous trial, and their interaction. The subject variable was entered as a random intercept. Also, trial number nested in block number was entered as a random slope, but this model was close to a singular fit, so the random slope term was dropped from the model. To address H3, a multilevel logistic regression was run, where the outcome was the binary trust choice of the current trial. The predictors were feedback (only cooperation trials: “better‐than‐expected” vs. “as‐expected”) of the previous trial, FLT of the previous trial, and their interaction. The subject variable was entered as a random intercept. Also, trial number nested in block number was entered as a random slope, but this model was close to a singular fit, so the random slope term was dropped from the model. To address H4, a simple linear regression was run, where the outcome variable was the frequency of trust choices across the EMA‐TGs and the predictor was the frequency of trust choices across all laboratory TG trials. It was preregistered that H5 and H6 would be tested with MLM, respectively. However, this was not applicable to the level of measurement of the collected data. Hence, multiple linear regression models were run, where the outcome variable was the mean trust rate during EMA and the predictors were first, FCB, second, MFT power difference between trials of unexpected defection (individualistic trustee behavior) and expected defection feedback, and third, FLT power difference between trials of unexpected and expected cooperation feedback.

## 3 Results

### 3.1 Descriptive Results

The sample to answer the hypotheses involving laboratory variables only consisted of 55 participants (13 male, mean age = 27.25, SD = 10.64). The sample to answer the combined hypotheses consisted of 54 participants (12 male, mean age = 27.31, SD = 10.73). Their SVO° ranged from 11.62° to 45.00° (mean = 35.07°, SD = 8.00°, c.f. Figure S4). Of those, 13% fell into the category “Individualism” (12.04° < SVO° < 22.45°), whereas all others (87%) fell into the category “Prosociality” (22.45° < SVO° < 57.15°). None fell into the categories “Altruism” (SVO° > 57.15°) or “Competitiveness” (SVO° < −12.04°). The mean and median of the EMA response rates were 62% (SD = 18%) ranging from 21% to 96% (out of 24 prompts). A total 80 of 4 EMA prompts with social interactions were analyzed. The mean of the trust rates in the EMA was 83% (SD = 19%) and negatively skewed (−1.87).

### 3.2 Manipulation Check

The manipulation check was significant, *t*(119) = 13.626, *p* < 0.001 (mean difference of 1.45 between conditions), indicating that biasing the trustees' initial trustworthiness worked.

### 3.3 Effects of Blockwise Manipulation of Reputational Prior and Trustee Behavior on Trust Decisions

**FIGURE 2:**
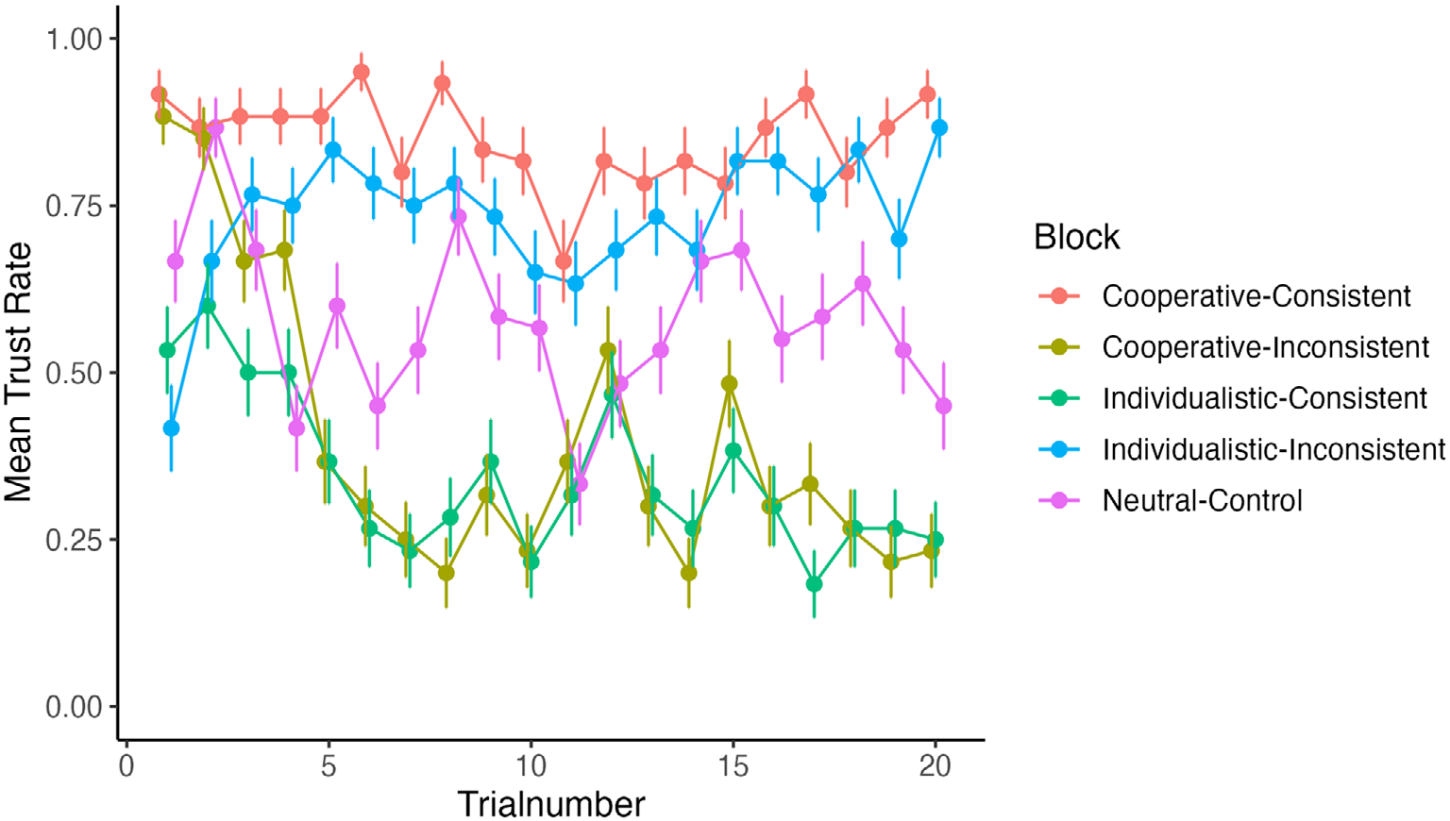
Trust choice rate over trials split by block. Error bars indicate standard error of the mean. To enhance credibility, 20% of the trials within a block (except Neutral‐Control) deviated from strictly cooperative trustee behavior (Cooperative‐Consistent, Individualistic‐Inconsistent) or from strictly individualistic trustee behavior (Individualistic‐Consistent, Cooperative‐Inconsistent). Its effect can, , be observed in trial 12 in the Cooperative‐Inconsistent and Individualistic‐Consistent block, where the trust rates increase in blocks of otherwise 80% individualistic trustee behavior.

Participants quickly adjusted their decision policy to the trustees' return rates in the “inconsistent” blocks within less than ten trials, whereby the pattern of adjustment closely resembled a logarithmic growth or decay process (see Figure 2). Descriptively, the within‐block adjustment was more pronounced across participants if a “cooperative” reputational prior was followed by “individualistic” behavior (“cooperative‐inconsistent”) than if an “individualistic” reputational prior was followed by “cooperative” behavior (“individualistic‐inconsistent”). To investigate this relationship, we fitted a simple linear model with the outcome variable being the mean trust rate across participants and the predictor variables being block and trial number. Herby, we only used inconsistent blocks and inverted the trial number of the “cooperative‐inconsistent” block to account for the inverse slope orientation. Comparing the two slopes yielded a significant interaction term of block and trial number (*F* (1, 36) = 5.25, *p* = 0.028, *β* = 0.414), such that the decline of the “cooperative‐inconsistent” slope was steeper (estimated marginal trend: (*b* = 0.024, SE = 0.005, 95% CI [0.013, 0.034])) than the incline of the “individualistic‐inconsistent” slope (estimated marginal trend: *b* = 0.007, SE = 0.005, 95% CI [−0.003, 0.018]).

### 3.4 Effect of Fronto‐central Beta Power on Trust Decisions

Contrary to expected in H1, a higher beta‐power during decision generation did not significantly increase the odds (OR = 1.00, 95% CI [0.98, 1.01], *p =* 0.677) of a trust choice in the subsequent choice execution. Descriptively, beta power even slightly decreased the likelihood of a trust choice (see Table 1).

**TABLE 1:** Mixed logistic regression results using trust choice of trial_n_ in laboratory trust game as the outcome (H1).

	*χ* ^2^	df	*p*	*b*	SE (*b*)	Odds ratio (OR)	OR CI LL	OR CI UL
Intercept	6.492	1	0.011	1.109	0.435	3.030	1.291	7.108
Beta power at FCz in trial_n_	0.174	1	0.677	−0.003	0.007	0.997	0.984	1.011

*Note:* *Nagelkerke's R* ^2^ = 0.178, CI, 95% confidence interval; LL, lower level; UL, upper level; model specification: trust choice in trial_n_ ~ beta power at FCz in trial_n_ + (1|subject) + (1 + trialnumber|blocknumber).

### 3.5 Effects of Trial‐by‐trial Feedback and Midfrontal Theta Power on Trust Decisions

**FIGURE 3:**
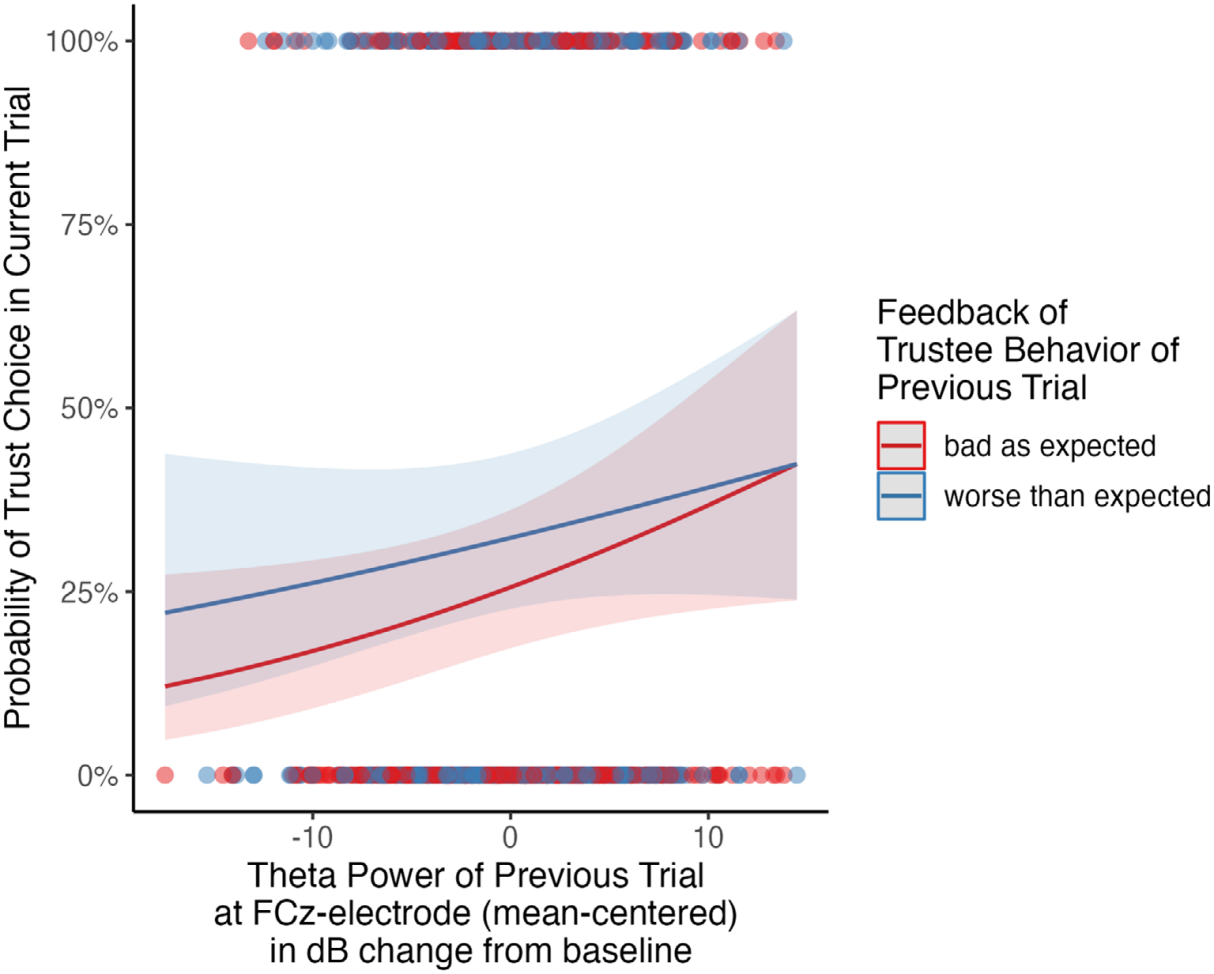
Predicted values of the mixed logistic model concerning the trust choice in trial_n_ by midfrontal theta power (FCz) Split by feedback of trustee behavior in trial_n‐1_. Shaded errors indicate 95% confidence intervals.

Contrary to expected in H2, the interaction between the feedback in trial_n‐1_ (“worse than expected” vs. “bad as expected” by reputational prior) and MFT during feedback was not significant for trust choices (*χ* ^2^(1) = 0.40, *p* = 0.529). Also, an MFT increase of 1 dB from baseline in response to negative feedback in trial_n‐1_ significantly increased the odds of a trust choice in a subsequent trial by 5.4% ((OR = 1.054), 95% CI [1.003, 1.107], *p* = 0.036). This main effect was independent of the previous trial having been “bad as expected” or “worse than expected” (see Figures 3 and 5; Table 2).

**TABLE 2:** Mixed logistic regression results using trust choice of trial_n_ in laboratory trust game as the outcome (H2).

	*χ* ^2^	df	*p*	*b*	SE (*b*)	Odds ratio (OR)	OR CI LL	OR CI UL
Intercept	17.653	1	< 0.001	−1.067	0.254	0.344	0.209	0.566
Feedback in trial_n‐1_	3.360	1	0.067	0.328	0.179	1.388	0.978	1.972
Theta Power at FCz	**4.389**	**1**	**0.** **036**	**0.052**	**0.025**	**1.054**	**1.003**	**1.107**
Feedback in trial_n‐1_ × Theta Power at FCz	0.397	1	0.529	−0.023	0.036	0.978	0.911	1.049

*Note:* Feedback in trial_n‐1_ (1 = worse than expected, 0 = bad as expected), bold values are significant at *p* < 0.05, *Nagelkerke's R* ^*2*^ = 0.016, CI, 95% confidence interval; LL, lower level; UL, upper level; model specification: trust choice in trial_n_ ~ feedback in trial_n‐1_ × theta power at FCz + (1|subject).

**FIGURE 4:**
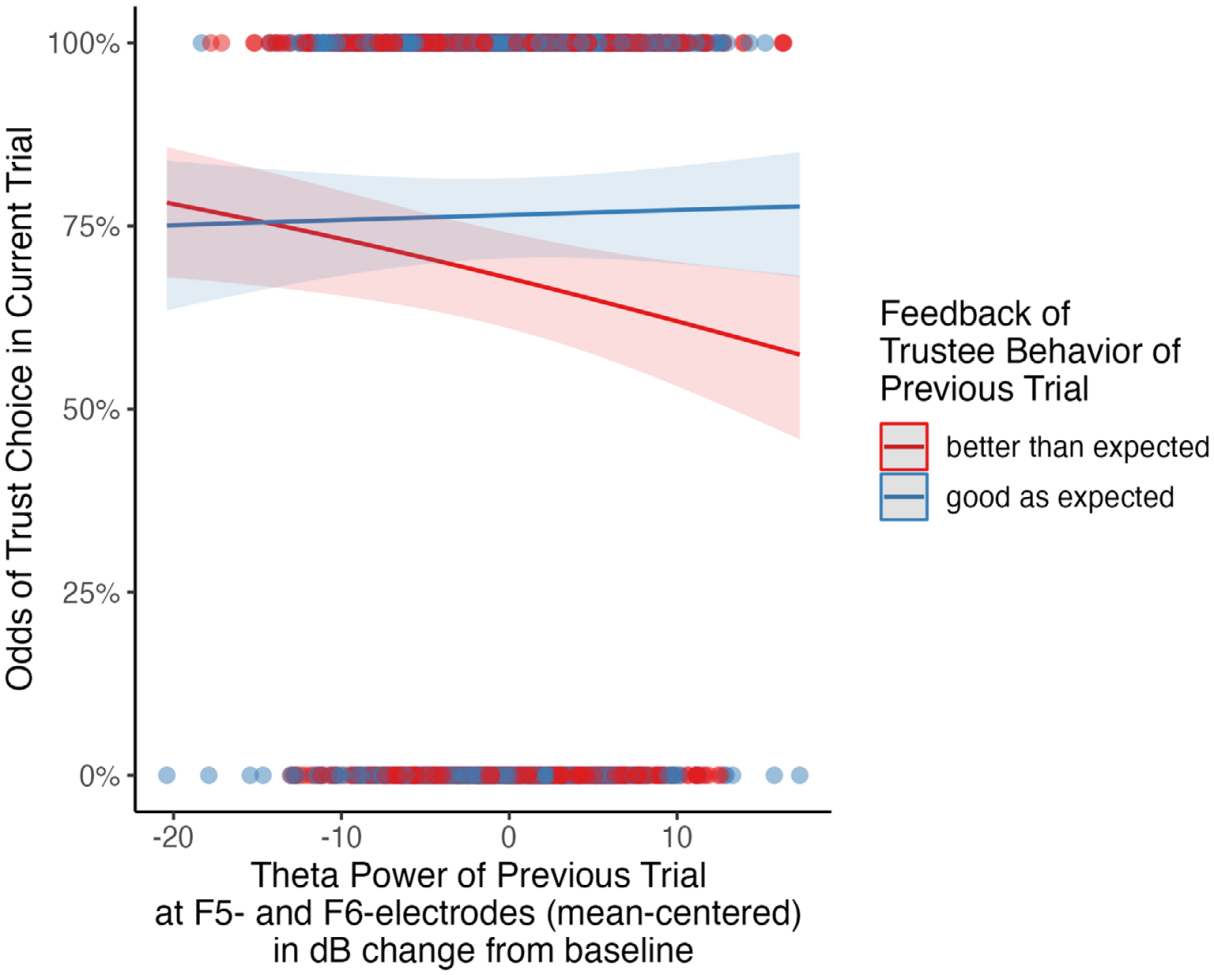
Predicted values of the mixed logistic model regarding the probability of trust choice in trial_n_ by frontolateral theta power (FC5, FC6) Split by feedback of trustee behavior in trial_n‐1_. Shaded errors indicate 95% confidence intervals.

**FIGURE 5:**
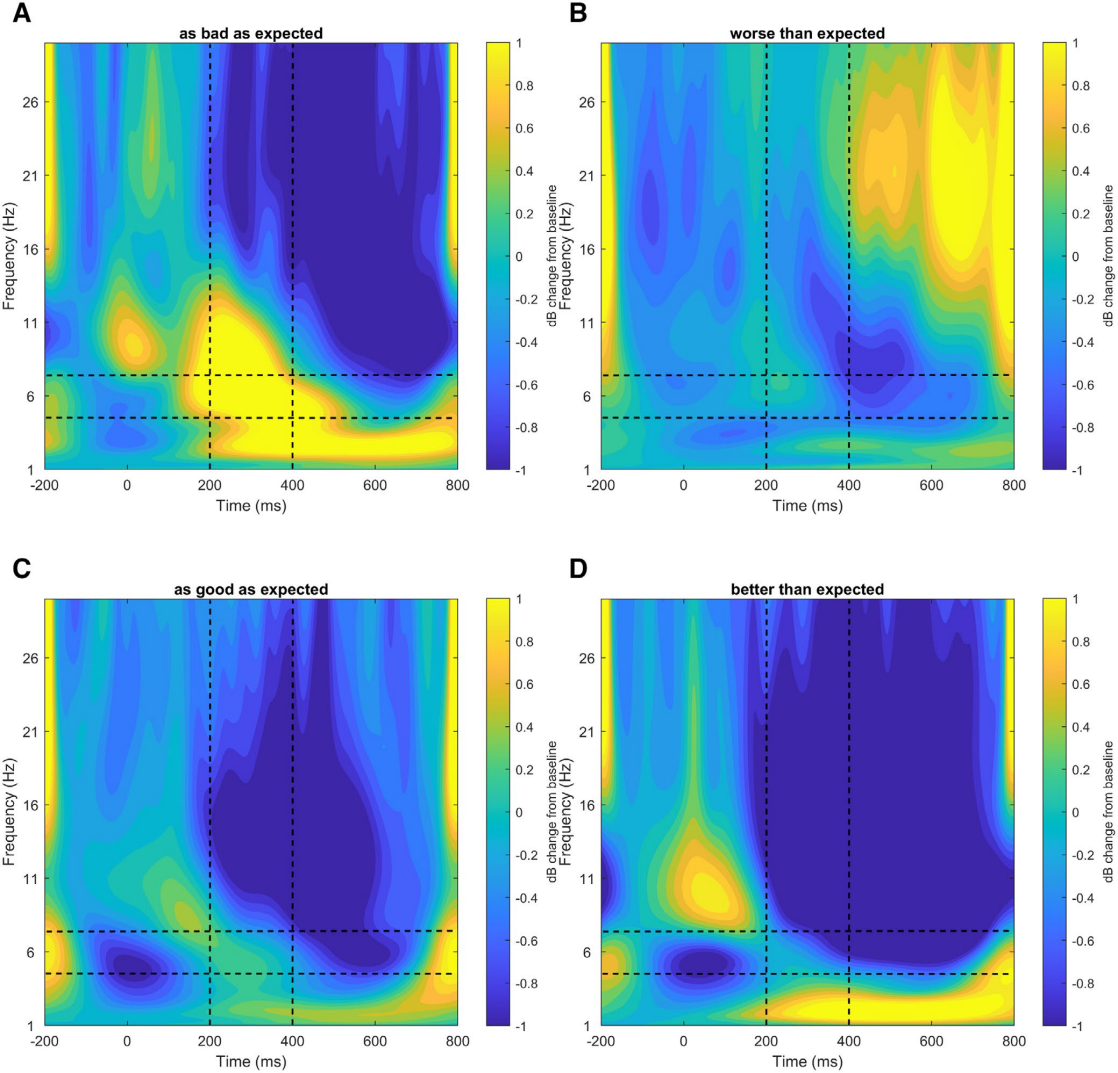
Event‐related spectral power at the FCz‐electrode (A, B) and the FC5, FC6‐electrodes (C, D) during Trust Game feedback split by feedback type. Dashed black lines indicate the theta band (200–400 ms, 4–8 Hz).

Contrary to expected in H3, no significant interaction effect between feedback and FLT was found to predict trust choice (*χ* ^2^ (1) = 3.60, *p* = 0.580). In fact, if feedback in trial_n‐1_ was “better than expected”, a higher FLT even decreased the odds of a trust choice in trial_n_ descriptively. The main effect of feedback was significant: If trial_n‐1_ was “as good as expected”, it significantly increased the odds of a trust choice in trial_n_ by 54.2% (OR = 1.542, 95% CI [1.325, 1.796], *p* < 0.001) compared to feedback being “better than expected”. The main effect of FLT was also significant: A 1 dB increase in FLT from baseline in response to positive feedback in trial_n‐1_ decreased the odds of a trust choice in trial_n_ significantly by 2.6% (OR = 0.974, 95% CI [0.954, 0.995], *p* = 0.014). However, both main effects were driven by the descriptive interaction between FLT and feedback (see Figures 4 and 5; Table 3).

**TABLE 3:** Mixed logistic regression results using trust choice of trial_n_ in experimental Trust Game as the outcome (H3).

	*χ* ^2^	df	*p*	*b*	SE (*b*)	Odds ratio (OR)	OR CI LL	OR CI UL
Intercept	23.776	1	< 0.001	0.748	0.153	2.112	1.564	2.853
Feedback in trial_n‐1_	**31.188**	**1**	**< 0.001**	**0.433**	**0.078**	**1.542**	**1.325**	**1.796**
Theta Power at F5, F6	**6.013**	**1**	**0.** **014**	**−0.026**	**0.011**	**0.974**	**0.954**	**0.995**
Feedback in trial_n‐1_ × Theta Power at F5, F6	3.599	1	0.058	0.030	0.016	1.030	0.999	1.062

*Note:* Feedback in trial_n‐1_ (1 = good as expected, 0 = better than expected), bold values are significant at *p* < 0.05, *Nagelkerke's R* ^2^, 0.015; CI, 95% confidence interval; LL, lower level; UL, upper level; model specification: trust choice in trial_n_ ~ feedback in trial_n‐1_ × theta power at F5, F6 + (1|subject).

### 3.6 Predicting Daily Trust Decisions

**FIGURE 6:**
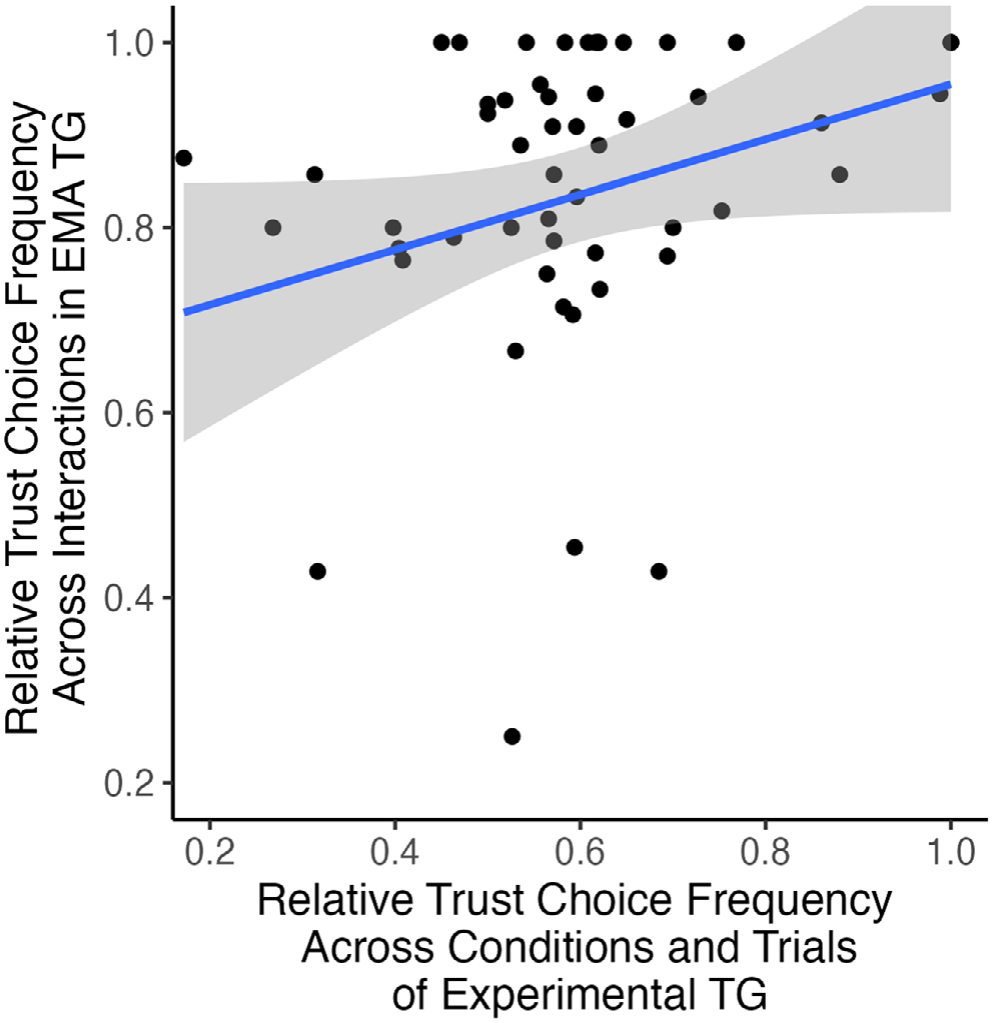
Regression of relative trust choice frequency in experimental versus ecological assessment trust game. Shaded area indicates 95% confidence intervals.

A higher trust rate in the laboratory TG did not significantly predict more trust choices in the EMA‐TG (*F* (1, 52) = 3.66, *p* = 0.061, *β* = 0.256, adjusted *R* ^*2*^ *= 0*.048), contrary to expected in H4. Descriptively, however, there was a trend in the expected direction (see Figure 6). This finding remained consistent after controlling for outliers by using a Cook's distance threshold of > 4/N–0.005, which also improved model fit slightly (*F* (1, 47) = 3.51, *p* = 0.067, *β* = 0.264, adjusted *R* ^2^ *= 0*.050). For exploratory purposes, we split the experimental data by block and found that the “individualistic‐inconsistent” “cooperative‐consistent” and the “neutral‐consistent” blocks were significant after removing outliers based on Cook's distance > 4/N (*p* < 0.05; see Figure S7).

Contrary to expected in H5, beta power did not significantly predict trust choice rates in the EMA‐TG (*b* = 0.02, SE = 0.03, *p* = 0.577). Also, contrary to expected in H6, neither a higher MFT difference during negative feedback (“bad as expected”—“worse than expected”, *b* = 0.01, SE = 0.01, *p* = 0.417), nor a higher FLT difference during positive feedback (“good as expected”—“better than expected”, *b* = 0.03, SE = 0.02, *p* = 0.122) was a significant predictor of the trust choice rate in the EMA‐TG (Table 4).

**TABLE 4:** Regression results using relative trust choice frequency across Interactions in ecological trust game as outcome and electrocortical potentials as predictors (H5, H6).

	*b*	SE (*b*)	*p*	CI LL	CI UL	*β*
Intercept	0.849	0.041	< 0.001	0.767	0.932	
FCB power at FCz (mean across experiment)	0.019	0.034	0.577	−0.049	0.088	0.077
Mean MFT power difference (bad as expected—worse than expected)	0.009	0.012	0.417	−0.014	0.033	0.112
Mean FLT Power difference (good as expected—better than expected)	0.028	0.018	0.122	−0.008	0.064	0.216

*Note:* Adjusted *R* ^2^, 0.005; CI, 95% confidence interval; FCB, fronto‐central beta; FLT, fronto‐lateral theta; LL, lower level; MFT, midfrontal theta; SE (*b*), standard error of the mean; UL, upper level; model specification: relative trust choice frequency across interactions in ecological trust game ~ mean beta power at FCz across trials + mean theta power difference at FCz (bad‐as‐excepted—worse‐than‐expected) + mean theta power difference at F5, F6 (good‐as‐expected—better‐than‐expected).

## 4 Discussion

The first aim of the current study was to determine how positive and negative prior reputations that are inconsistent with partners' behavior influence trust decisions differentially. Thus, we investigated how the integration of reputation, which is inconsistent or consistent with actual TG return rates, is reflected in neurophysiological activity and how these changes may influence trust decisions. The second aim was to determine whether and how laboratory TG behavior and associated neurophysiological activity can inform daily trust decisions. To do so, we first recorded EEG and trust decisions while participants played an iterated TG with four anonymous partners who were introduced as either cooperative or individualistic. Partners' return rates either confirmed or disconfirmed these reputational priors. We then used behavioral and neurophysiological correlates (FCB, MFT, FLT) to predict trust choices in daily interactions using EMA.

Participants quickly adjusted their decision behavior to the trustees' return rates in “inconsistent” blocks within less than ten trials. We found that this adjustment was more pronounced if a “cooperative” reputational prior was followed by “individualistic” behavior than if an “individualistic” reputational prior was followed by “cooperative” behavior by comparing the slopes. Stated plainly, participants were more inclined to “unlearn” trustworthiness than to learn that the partner is trustworthy despite a bad reputation. This result extends the findings of Delgado et al. (2005), who showed that participants were more willing to trust partners with a “good” reputation, irrespective of the reciprocity rate (which was 50% for all trustees) in a TG with interleaved partner interactions (24 per trustee). The authors suggested that a positive reputation might have biased the participants to ignore negative feedback, hindering the integration of negative partner feedback. On the behavioral level, we now demonstrate that this might only, if at all, be the case for negative prior information and not for positive prior information. However, more TG trials would be required to confirm this interpretation. Our findings corroborate those of Chang et al. (2010): across different reinforcement learning models, they also found that negative trustee behavior (in 15 repeated TG interactions) is more readily integrated (with a substantially higher learning rate) than cooperative trustee behavior using facial trustworthiness as prior initialization. These findings are also in line with the general idea of the loss aversion tendency proposed by prospect theory (Tversky and Kahneman 1992).

This tendency might also be reflected by electrocortical correlates (H3): On the trial level, higher FLT in response to feedback being “better than expected” descriptively predicted lower odds of a trust choice in the subsequent trial (H3). This corroborates the previously suggested inhibitory control function theta in general (Cohen 2014a) and FLT in particular (Krämer et al. 2013). As such, FLT may be responsible for maintaining suspicion even if a negative reputation is disconfirmed by positive partner behavior. This function of the FLT has already been discussed in other studies investigating general stimuli‐action conflicts (Krämer et al. 2013; Martínez‐Molina et al. 2024). Our findings did not corroborate the hypothesized interaction between MFT and feedback type (H2), since we observed that elevated MFT promoted trust in subsequent trials, irrespective of reputation consistent or inconsistent partner behavior. This contradicts the notion that MFT signals a heightened need for control (as suggested by Cavanagh and Shackman 2015; Cavanagh et al. 2010) that translates to immediate action in situations of conflicting social information. It also suggests that the MFT's role in trust decision making might be more general, potentially facilitating the avoidance of precipitate distrust decisions in general, rather than adjusting behavior based on feedback conformity with reputational priors.

Contrary to previous results (Declerck et al. 2013; Wang et al. 2021), we did not find that higher FCB during decision generation increased the general odds of a trust choice (H1). This might be the case, since we deviated from the design by Wang et al. (2021): First, we did not split the data by previous feedback, although they found that the feedback of a previous trial moderated the predictive effect of beta power on individual trust choices on a current trial. In their study, beta power could only predict trust choices in trials that were preceded by cooperative trustee feedback. Second, we restricted the electrode of interest (FCz) beforehand, whereas they used electrodes discriminating trust‐ and distrust choices, yielding five frontal positions. And third, they found that predictions by beta power improved when individual trust rates are low. The current study found slightly elevated trust rates across experimental conditions (e.g., 58% in the control condition with chance level reciprocity) which may have thus impaired our predictions. Disregarding methodological differences, cognitive control reflected by beta power cannot be ubiquitously assumed to facilitate trust, especially if it favors the maintenance of a behavioral policy that is informed by a misleading reputational prior. Then, cognitive control may even hinder “correct” trust choices. Further studies should investigate this possibility.

Furthermore, laboratory TG behavior did not significantly predict daily trust levels, although there was a descriptive linear trend among the participants with balanced trust rates (H4). This relationship was weakened by ceiling effects, indicated by substantial, but still acceptable negative skewness (−1.87; Curran et al. 1996), particularly due to participants who only chose to trust their daily interaction partners (24%). However, the linear trend was robust to controlling for outliers. The skewness may reflect a natural tendency to associate with trustworthy individuals. Our data also align with Weiss et al. (2021), who noted that experimental TG behavior could predict trust levels for distant but not close partners. Experimental blocks where trustee behavior was cooperative in 80% or 50% of trials predicted everyday trust levels significantly, irrespective of prior reputation. This suggests that subjects may expect at least chance levels of reciprocity in everyday interactions. Since subjects quickly adjusted their trust decision behavior to actual partner reciprocity, the effect of prior reputation on overall trust rates in the block was small. Yet, to “forget about” a negative prior faster was associated with increased trust levels in everyday interactions. Although our results are statistically consistent with those of Glaeser et al. (2000) and Weiss et al. (2021), they do not unequivocally support rejecting the ecological validity of the TG.

Since FCB was not a significant predictor of trust decisions in the laboratory TG, it also did not inform daily trust levels (H5). MFT and FLT did not interact with the feedback type (consistent vs. inconsistent) to inform trust decisions in the laboratory TG. Therefore, it cannot be assumed that these signals are ubiquitous markers of flexible behavioral adjustment rendering cooperation more successful. Hence, their difference between consistent and inconsistent trials did not inform daily trust decisions, either (H6). However, the interaction of FLT with feedback type (good as expected vs. better than expected) approached significance for the laboratory trust decisions. FLT was also the best predictor compared to FCB and MFT. This parallels recent results by Dell'Acqua et al. (2024) who found that the reward positivity in response to social rewards in the laboratory moderated the link between positive events of social support and higher positive affect in everyday life. Although we used theta power instead of an ERP, we also investigated the differential activation of feedback that is better than expected. Future studies may thus rather focus on robust ERPs instead of frequency bands to translate laboratory findings to everyday experience and behavior. Also, neural responses to positive compared to negative expectation violations may be better predictors of daily trust than negative ones.

### 4.1 Limitations

The a priori power calculation was primarily based on the hypothesis that aimed to predict trust decisions by electrocortical correlates, as we expected this to be the smallest effect. At the time of preregistration, there were no studies available investigating similar research questions with EEG. However, simulation‐based approaches, or the formulation of more comprehensive statistical models, would have enabled better estimates of power, or required sample size. Hence, we cannot preclude that our study was underpowered.

Considering the experimental design, the effect of the reputational prior might not have lasted long enough to alter theta processes with respect to behavior in all 20 trials, such that the expected trial‐level interactions could not be detected. Assuming MFT to be an indicator of cognitive control, the paradigm might not have required cognitive control to override behavioral tendencies to make prior‐consistent choices in all trials, but only within the first 5–10 (see Figure 2). The brief three‐day sampling period and average participant response rate of 15 EMA‐TG prompts resulted in a narrow interaction range. Extending the sampling period could have captured interactions with a higher variance of partner trustworthiness and could have thus mitigated ceiling effects. Attempts to use electrocortical potentials to predict daily trust choices were exploratory due to the absence of previous studies. Hence, the small and non‐significant findings of the laboratory research might have pre‐emptively challenged the hypotheses by combining the EEG findings with EMA.

Considering our feedback design, the visual structure of the feedback stimuli was different depending on the participant's trust decisions, so that distrust decisions yielded larger stimuli, because the trustee's behavior had to be revealed. This rendered the feedback stimulus more complex, and thus more salient, which has been shown to elicit an increased amplitude of the feedback‐related negativity (Pfabigan et al. 2015, 2019). This may also influence MFT, since the theta band also contains N2 activation components (Cavanagh et al. 2012; Rodrigues et al. 2022). However, feedback conditions after cooperation and non‐cooperation were not directly tested against each other. Nevertheless, some blocks yielded more cooperation decisions by design. Still, our hypotheses and design required feedback of the trustees’ behavior, irrespective of the participants’ previous decision, so we approved of this limitation. Future studies should eradicate the difference in stimulus complexity between conditions completely.

### 4.2 Conclusion

This study examined the integration of consistent and inconsistent reputational priors with actual partner behavior in a Trust Game at the behavioral and neural levels. In summary, participants adjusted their decision‐making based on inconsistent trustee behavior within a few trials, showing a stronger tendency to unlearn trustworthiness than to develop trust despite negative reputations. Theoretically, this pattern aligns well with previous computational models and loss aversion. Neural correlates suggest that processes underlying fronto‐lateral theta may maintain this distrust when partner behavior exceeds reputation‐based expectations, serving an inhibitory control function. Descriptively, there was an association between experimental levels and daily trust levels, especially for reputation‐consistent experimental blocks, supporting the validity of the Trust Game in naturalistic settings. Since this study did not find a frequency band predicting daily trust levels, we suggest that future studies also use ERPs of positive social expectation‐violations to inform daily trust decisions as moderators.

## Author Contributions

**Kilian Stenzel:** conceptualization, data curation, formal analysis, investigation, methodology, project administration, resources, software, validation, visualization, writing – original draft, writing – review and editing. **Martin Weiß:** conceptualization, data curation, formal analysis, funding acquisition, investigation, methodology, project administration, resources, software, supervision, visualization, writing – review and editing. **Grit Hein:** conceptualization, funding acquisition, methodology, resources, supervision, writing – review and editing.

## Disclosure

*Use of AI generated content* ( *AIGC* ) *and tools*: During the preparation of this work, the authors used ChatGPT 3.5 and DeepL Write in order to improve the style and grammar of the final manuscript. After using this tool/service, the authors reviewed and edited the content as needed and took full responsibility for the content of the publication.

## Conflicts of Interest

The authors declare no conflicts of interest.

## Supporting information


**Data S1.** Supporting Information.

## Data Availability

The data and code underlying this article will be shared on reasonable request to the corresponding authors.
