# Supplementary material for: A Negative Reputation Reduces Trust Despite Trustworthy Behavior
Source: Psychophysiology. 2025 Jul 1;62(7):e70102. doi: 10.1111/psyp.70102 (PMC12214295; doi:10.1111/psyp.70102)
Supplement: Supplementary file 1 — Data S1. Supporting Information. [file PSYP-62-e70102-s001.pdf]

Supplementary Information for "A negative reputation reduces trust despite trustworthy behavior" (Kilian Stenzel, Martin Weiß, Grit Hein, 2025)

List of Figures

Figure S1. Induction of the Reputational Prior 3

Figure S2. Trustworthiness Rating 4

Figure S3. Exemplary SVO-item (of 15). 4

Figure S4: Influential Observations by Cook's Distance 9

Figure S5: Comparison of Regression Slope before and After Removal of Influential Data Based on Cook's Distance 11

Figure S6: Density Plots of Outlier Characteristics 12

Figure S7. H4 Exploratory Analysis: Simple Linear Regressions of Relative Trust Choice Frequency in Laboratory vs Ecological Trust Game Split by Block 14

List of Tables

Table S1. Counterbalancing 3

Table S2. Mean Rate of Trust Choice Within Block Across Participants 6

Table S3. Mean Rate of Trust Choice in $Trial_n$ by Condition (Feedback in $Trial_{n-1}$) 6

Table S4. Effects of Simple Linear Model with Outcome Mean Trust Choice Across Participants and Predictors Trial Number and Inconsistent Conditions 6

Table S5. H1: Mixed Logistic Regression Results Using Trust Choice in Laboratory Trust Game as the Outcome 7

Table S6. H2: Mixed Logistic Regression Results Using Trust Choice in Laboratory Trust Game as the Outcome. 7

Table S7. H3: Mixed Logistic Regression Results Using Trust Choice in Laboratory Trust Game as the Outcome. 8

Table S8. H4: Regression Results Using Relative Trust Choice Frequency Across Interactions in Ecological Trust Game as Outcome and Mean Trust Rate in Laboratory Trust Game as Predictor 8

Table S9. H4: Comparison of Regression Results after Outliers Removed Based on Cook's Distance (> 4/N) and Cook's Distance (> 4/N – 0.005) Using Relative Trust Choice Frequency Across Interactions in Ecological Trust Game as Outcome and Mean Trust Rate in Experiment as Predictor 10

Table S10: Comparison of Regression Results after Outliers Removed Based on Cook's Distance (> 4/N) Using Relative Trust Choice Frequency Across Interactions in Ecological Trust Game as Outcome and Mean Trust Rate in Experiment as Predictor 13

Table S11. H5, H6: Regression Results Using Relative Trust Choice Frequency Across Interactions in Ecological Trust Game as Outcome and Electrocortical Potentials as Predictors 15

Table S12. H6: Regression Results Using Relative Trust Choice Frequency Across Interactions in Ecological Trust Game as Outcome and Electrocortical Potentials as Predictors........ 15

Table S13. Descriptives: Mean Response Time for Trust Game Choice by Block 16

Table S14. H7: Mixed Linear Regression Results Using Response Time for Trust Choice Execution in Laboratory Trust Game as the Outcome 16

Table S15. H8: Mixed Linear Regression Results Using Response Time for Trust Choice in Laboratory Trust Game as the Outcome 17

Table S16. H9: Mixed Linear Regression Results Using Feedback Related Negativity as the Outcome 17

Table S17. H10: Mixed Linear Regression Results Using P2 as the Outcome........ 18

Methods

Design

Table S1. Counterbalancing

Order of appearance	Counterbalance 1	Counterbalance 2	Counterbalance 3	Counterbalance 4
1st	CC	II	CI	IC
2nd	II	CC	IC	CI
3rd	NC	NC	NC	NC
4th	IC	CI	CC	II
5th	CI	IC	II	CC

Note. Cooperative-consistent (CC), cooperative-inconsistent (CI), individualistic-consistent (IC), individualistic-inconsistent (II), neutral-consistent (control, NC).

Procedure

Figure S1. Induction of the Reputational Prior

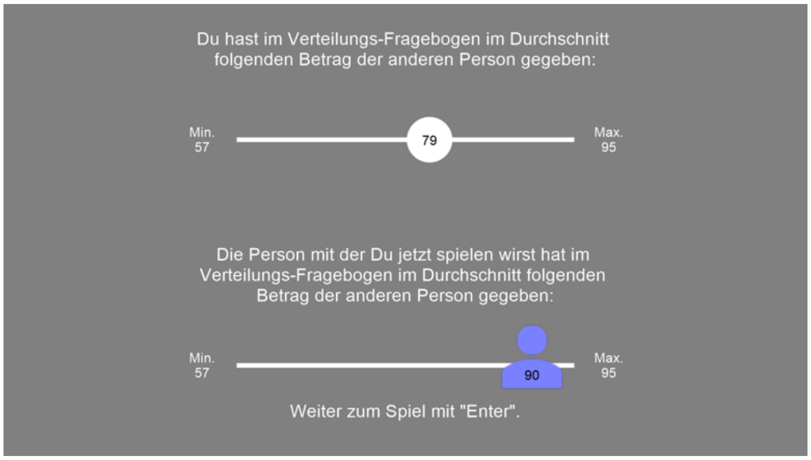

Note. The "induction of the reputational prior" displays two scales being anchored at the minimum ("57") and maximum ("95") average amount one could allocate in the SVO: Upper scale: Average number of points the subject gave to the other in the SVO previously taken indicated by an integer on a white circle being shifted on the scale according to the amount. Lower scale: Average amount of points the trustee gave to the other in the SVO indicated by an integer on a colored pictographic bust being shifted according to the amount. This example shows an induction of the cooperative reputational prior. SVO = Questionnaire of Social Value Orientation.

Figure S2. Trustworthiness Rating

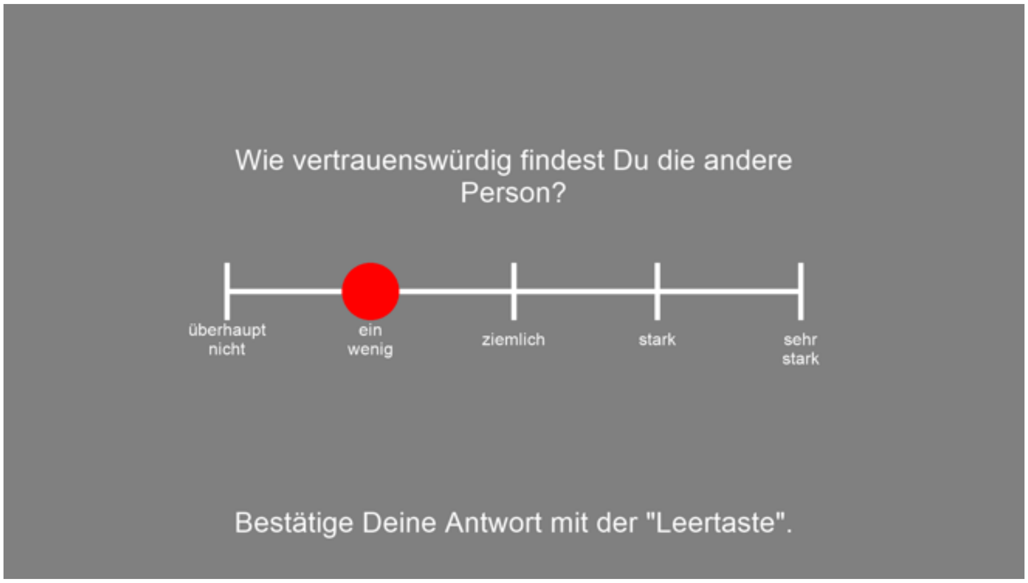

Note. Translated from German: (1) first row: “How trustworthy do you find the other person?”, (2) second row: “not at all”, “a little”, “fairly”, “strongly”, “very strongly”, (3) third row: “Confirm your answer with the space bar”

Figure S3. Exemplary SVO-item (of 15).

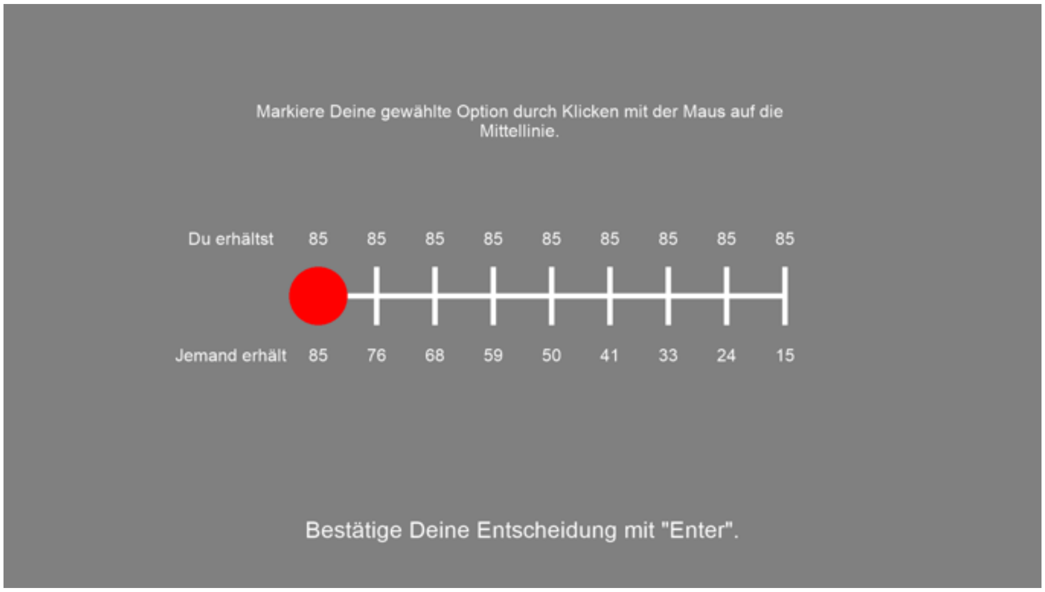

Note. Translated from German: (1) first row: “Mark your preferred option by clicking on the midline with your cursor” (2) second row: “You get”, (3) third row: “Someone gets”, (4) fourth row: “Confirm your choice by pressing “Enter”.

Exploratory EMA Variables

Exploratory variables collected during the EMA-survey contain contextual information about the latest interaction partner and the interaction itself (in order of appearance): Subjective wellbeing (continuous slider, “I feel”, anchored at “very bad” and “very good”), context of interaction (single-choice, “private” vs. “work”), medium (single-choice, “presence”, “virtual - texting”, “virtual - talking”), observers (single-choice, “alone” vs. “group”), pleasantness of interaction rated on a seven-point Likert scale (“How pleasant was then interaction?”, anchored at “very unpleasant” (1) and “very pleasant” (7)), gender (single-choice), guessed age (integer), type of relationship (multiple-choice), target initials or nickname (text string, to adjust for repeated interactions with the same person). Also, a self-report measure of the trustee's trustworthiness rated on a relative five-point Likert scale (“How trustworthy does this person seem to you?”, anchored at “not at all” (1) and “very strongly” (5)) was recorded.

EEG Preprocessing

Peaks for N2 (FRN) were detected on electrode positions FCz and Fz in the time window from 200 – 400 ms (preregistered: 200 - 350 ms) according to Schuermann et al. (2012) for the mean signal of trials being “worse than expected” and “bad as expected” across participants. For N2 at FCz, the peak was at 267 ms and for N2 at Fz, the peak was at 261 ms. Then, a 40 ms window was wrapped around these peaks (cf. Rodrigues et al., 2022). Second, the peak for P2 was on the electrode position FCz in the time window from 100 – 300 ms for the mean signal of trials being “better than expected” and “good as expected” across participants according to Schuermann et al. (2012). Again, a 40 ms window was wrapped around this peak (cf. Rodrigues et al., 2022). ERPs were weighted because feedback had different frequencies due to the free-choice structure of the TG.

Supplementary Results

Behavioral

Table S2. Mean Rate of Trust Choice Within Block Across Participants

Block	Mean Trust Choice Rate in %	SD of Trust Choice Rate in %
Cooperative-Consistent	84.8	.19.3
Cooperative-Inconsistent	38.4	21.8
Individualistic-Consistent	33.9	24.6
Individualistic-Inconsistent	73.5	24.0
Neutral-Control	57.5	21.6

Table S3. Mean Rate of Trust Choice in $Trial_n$ by Condition (Feedback in $Trial_{n-1}$)

Condition: Feedback in $Trial_{n-1}$	Frequency Distrust Choices $Trial_n$	Frequency Trust Choices $Trial_n$	Mean Trust Choice Rate $Trial_n$ in %
bad as expected	318	120	27.4
better than expected	96	358	78.9
good as expected	57	430	88.3
worse than expected	305	148	32.7

Note. The conditions are realized on the trial level within a block. Hence, the conditions stem from different blocks.

Table S4. Effects of Simple Linear Model with Outcome Mean Trust Choice Across Participants and Predictors Trial Number and Inconsistent Conditions

	b	SE (*b*)	*p*	CI LL	CI UL	*β*
(Intercept)	0.148	0.062	0.022	0.023	0.273	-0.708
**Condition (Reference: Cooperative-Inconsistent)**	**0.510**	**0.087**	**< 0.001**	**0.333**	**0.687**	**1.417**
**Trialnumber (inverted for Cooperative-Inconsistent)**	**0.024**	**0.005**	**< 0.001**	**0.013**	**0.034**	**0.592**
**Condition x Trialnumber**	**-0.017**	**0.007**	**0.028**	**-0.032**	**-0.002**	**-0.414**

Note. Bold values are significant at $p < 0.05$, CI = 95% confidence interval, LL = lower level, UL = upper level, model specification: mean trust choice ~ condition * trialnumber

Main Hypotheses

Table S5. H1: Mixed Logistic Regression Results Using Trust Choice in Laboratory Trust Game as the Outcome

	χ^2	df	*p*	*b*	SE (*b*)	Odds Ratio (OR)	OR CI LL	OR CI UL
(Intercept)	6.492	1	0.011	1.109	0.435	3.030	1.291	7.108
Beta Power at FCz	0.174	1	0.677	-0.003	0.007	0.997	0.984	1.011

Note. Bold values are significant at $p < 0.05$, *Nagelkerke's* $R^2 = .178$, CI = 95% confidence interval, LL = lower level, UL = upper level, model specification: trust choice in $trial_n$ ~ beta power at FCz in $trial_n$ + (1|subject) + (1 + trialnumber | blocknumber)

Table S6. H2: Mixed Logistic Regression Results Using Trust Choice in Laboratory Trust Game as the Outcome.

	χ^2	df	*p*	*b*	SE (b)	Odds Ratio (OR)	OR CI LL	OR CI UL
(Intercept)	17.653	1	< 0.001	-1.067	0.254	0.344	0.209	0.566
Feedback in $trial_{n-1}$	3.360	1	0.067	0.328	0.179	1.388	0.978	1.972
**Theta Power at FCz**	**4.389**	**1**	**0.036**	**0.052**	**0.025**	**1.054**	**1.003**	**1.107**
Feedback in $trial_{n-1}$ X Theta Power at FCz	0.397	1	0.529	-0.023	0.036	0.978	0.911	1.049

Note. Feedback in $trial_{n-1}$ (1 = worse than expected, 0 = bad as expected), bold values are significant at $p < 0.05$, *Nagelkerke's* $R^2 = .016$, CI = 95% confidence interval, LL = lower level, UL = upper level, model specification: trust choice in $trial_n$ ~ feedback in $trial_{n-1}$ * theta power at FCz + (1|subject)

Table S7. H3: Mixed Logistic Regression Results Using Trust Choice in Laboratory Trust Game as the Outcome.

	χ^2	df	*p*	*b*	SE (*b*)	Odds Ratio (OR)	OR CI LL	OR CI UL
(Intercept)	23.776	1	< 0.001	0.748	0.153	2.112	1.564	2.853
**Feedback in trial$_{n-1}$**	**31.188**	**1**	**< 0.001**	**0.433**	**0.078**	**1.542**	**1.325**	**1.796**
**Theta Power at F5, F6**	**6.013**	**1**	**0.014**	**-0.026**	**0.011**	**0.974**	**0.954**	**0.995**
Feedback in trial$_{n-1}$ X Theta Power at F5, F6	3.599	1	0.058	0.030	0.016	1.030	0.999	1.062

Note. Feedback in trial$_{n-1}$ (1 = good as expected, 0 = better than expected), bold values are significant at $p < 0.05$, *Nagelkerke's* R^2 = .015, CI = 95% confidence interval, LL = lower level, UL = upper level, model specification: trust choice in trial$_n$ ~ feedback in trial$_{n-1}$ * theta power at F5, F6 + (1|subject)

Table S8. H4: Regression Results Using Relative Trust Choice Frequency Across Interactions in Ecological Trust Game as Outcome and Mean Trust Rate in Laboratory Trust Game as Predictor

	b	SE (*b*)	*p*	CI LL	CI UL	*β*
(Intercept)	0.657	0.095	< 0.001	0.466	0.848	
Mean Trust Rate (Laboratory Trust Game)	0.298	0.156	0.061	-0.015	0.610	0.256

Note. Adjusted R^2 = .048*, SE (b)* = standard error of the mean, CI = 95% confidence interval*,* LL = lower level*,* UL = upper level, model specification: relative trust choice frequency across ecological trust game ~ mean trust rate in laboratory trust game

Figure S4: Influential Observations by Cook’s Distance

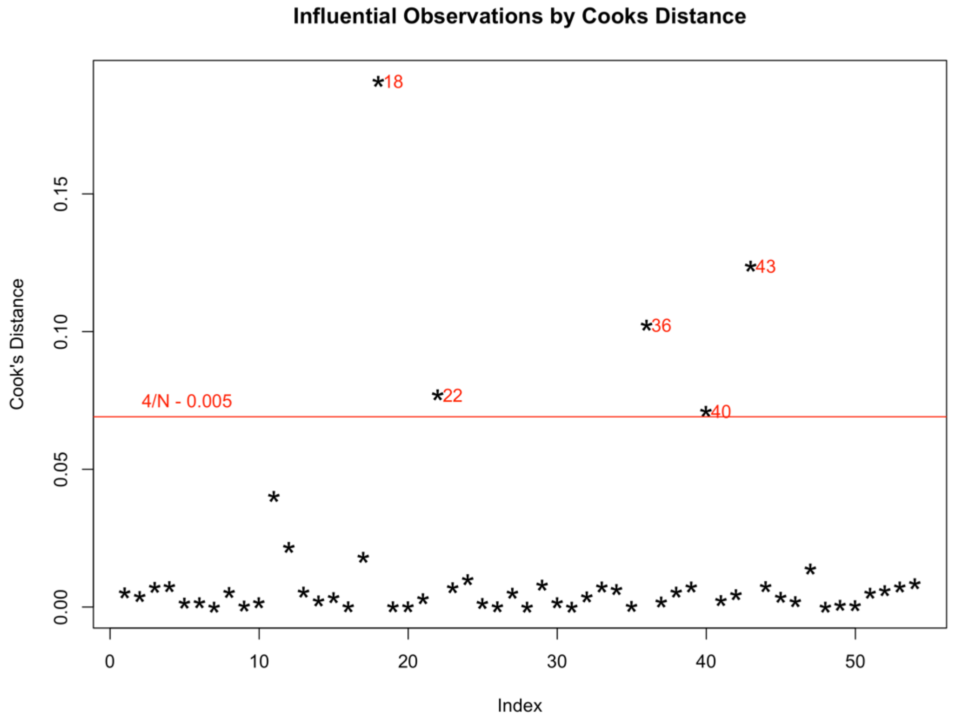

Note. Numbers indicate row numbers, not subject identifiers. Cook’s distance threshold was lowered marginally due to improved model fit (Table S8) and visual inspection.

Table S9. H4: Comparison of Regression Results after Outliers Removed Based on Cook's Distance (> 4/N) and Cook's Distance (> 4/N – 0.005) Using Relative Trust Choice Frequency Across Interactions in Ecological Trust Game as Outcome and Mean Trust Rate in Experiment as Predictor

Metric	Original	Outlier Removed (Cook's Distance > 4/N)	Outlier Removed (Cook's Distance > 4/N – 0.005)
Adj. R-squared	0.048	0.018	0.05
df1	1	1	1
df2	52	48	47
F	3.66	1.89	3.51
p	0.061	0.176	0.067
beta	0.256	0.195	0.264

Note. Cook's distance threshold was marginally lowered due to improved model fit and visual inspection of Figure S4.

Figure S5: Comparison of Regression Slope before and After Removal of Influential Data Based on Cook’s Distance

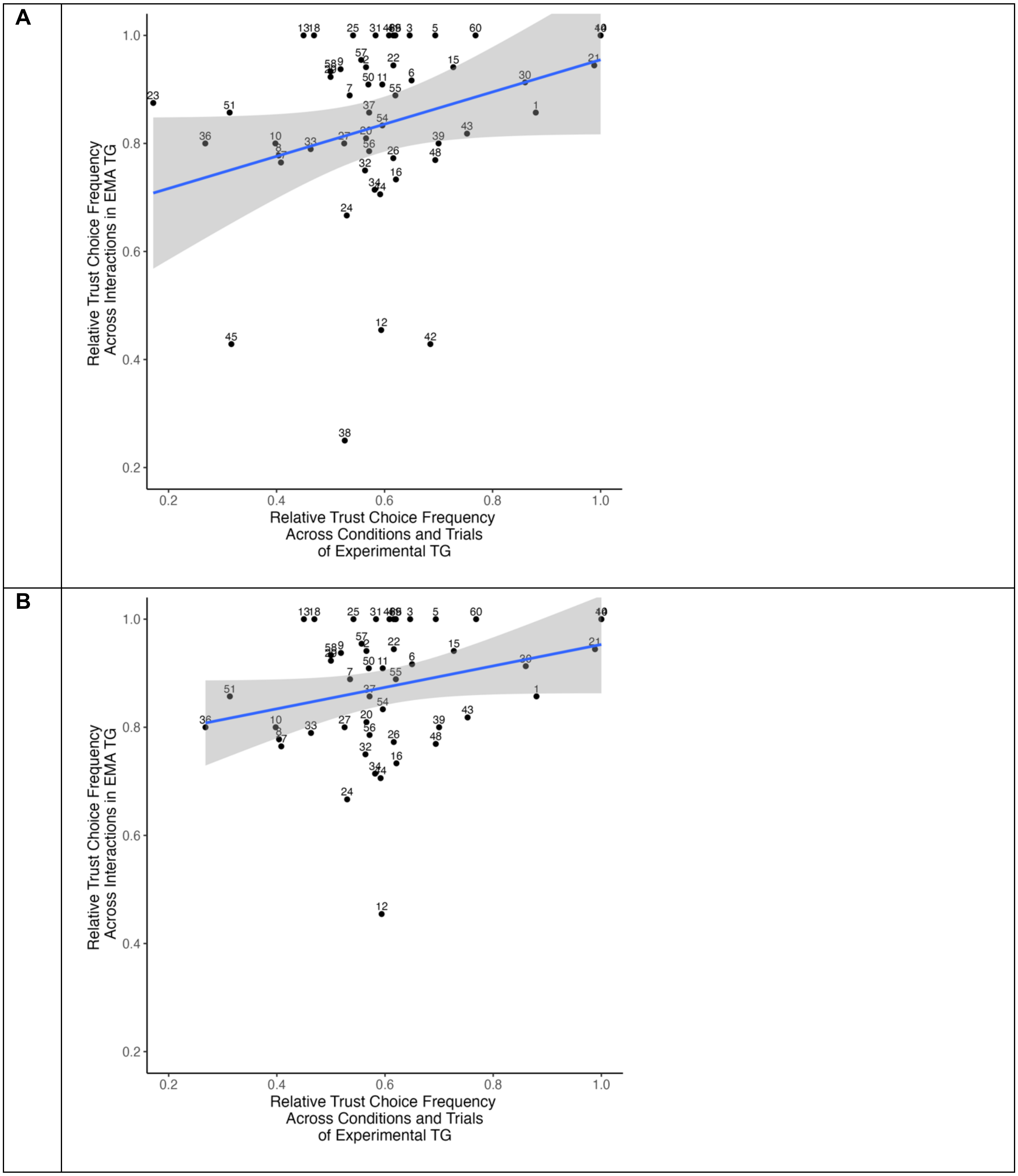

Note. Panel A: Original regression before correction for influential data, Panel B: Regression after correction for influential data (Cook’s Distance > 4/N – 0.005). Numbers indicate subject identifiers.

Figure S6: Density Plots of Outlier Characteristics

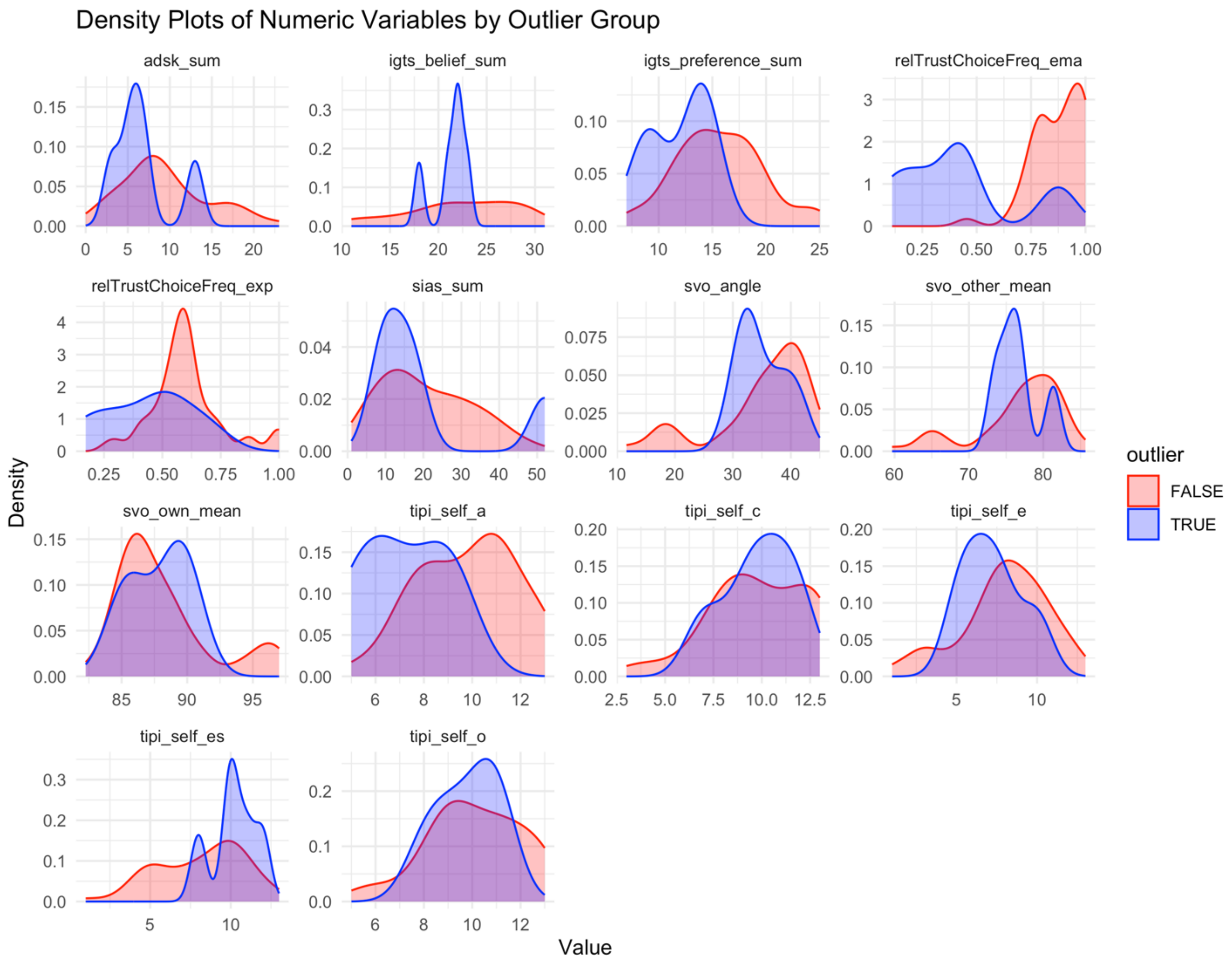

Note. adsk_sum = sumscore of depression inventory “Allgemeine Depressions-Skala – Kurzform”(ADS-K; Hautzinger et al., 2012; Hautzinger & Bailer, 1993), igts_belief_sum = “Inclusive General Trust Scale" - subscale belief, igts_preference_sum = sumscore "Inclusive General Trust Scale" - subscale preference, (IGTS; Jasielska et al., 2021; Yamagishi et al., 2015), relTrustChoiceFreq_ema = relative trust choice frequency during ecological assessment period, relTrustChoiceFreq = relative trust choice frequency during experiment, sias_sum = sumscore of Social Interaction Anxiety Scale (SIAS; Heimberg et al., 1992; Stangier et al., 1999), svo_angle = Social Value Orientation Index (Murphy et al., 2011), svo_other_mean = mean points allocated to other, svo_own_mean = mean points allocated to self, tipi = Ten-Item Personality Inventory (TIPI-G; Gosling et al., 2003; Muck et al., 2007) with subscales a = agreeableness, c = conscientiousness, e = extraversion, es = emotional stability, o = openness.

Table S10: Comparison of Regression Results after Outliers Removed Based on Cook's Distance (> 4/N) Using Relative Trust Choice Frequency Across Interactions in Ecological Trust Game as Outcome and Mean Trust Rate in Experiment as Predictor

Block	Metric	Original	Outlier Removed (Cook's Distance > 4/N)
Cooperative-Consistent	df1	1	1
Cooperative-Consistent	df2	52	48
Cooperative-Consistent	F	2.654	7.12
Cooperative-Consistent	**p**	**0.109**	**0.01**
Cooperative-Inconsistent	df1	1	1
Cooperative-Inconsistent	df2	52	48
Cooperative-Inconsistent	F	0.276	0.725
Cooperative-Inconsistent	p	0.601	0.399
Individualistic-Consistent	df1	1	1
Individualistic-Consistent	df2	52	48
Individualistic-Consistent	F	0.046	0.011
Individualistic-Consistent	p	0.831	0.919
Individualistic-Inconsistent	df1	1	1
Individualistic-Inconsistent	df2	52	48
Individualistic-Inconsistent	F	9.595	7.472
Individualistic-Inconsistent	**p**	**0.003**	**0.009**
Neutral-Control	df1	1	1
Neutral-Control	df2	52	49
Neutral-Control	F	4.386	4.074
Neutral-Control	**p**	**0.041**	**0.049**

Note. Bold rows indicate significance.

Figure S7. H4 Exploratory Analysis: Simple Linear Regressions of Relative Trust Choice Frequency in Laboratory vs Ecological Trust Game Split by Block

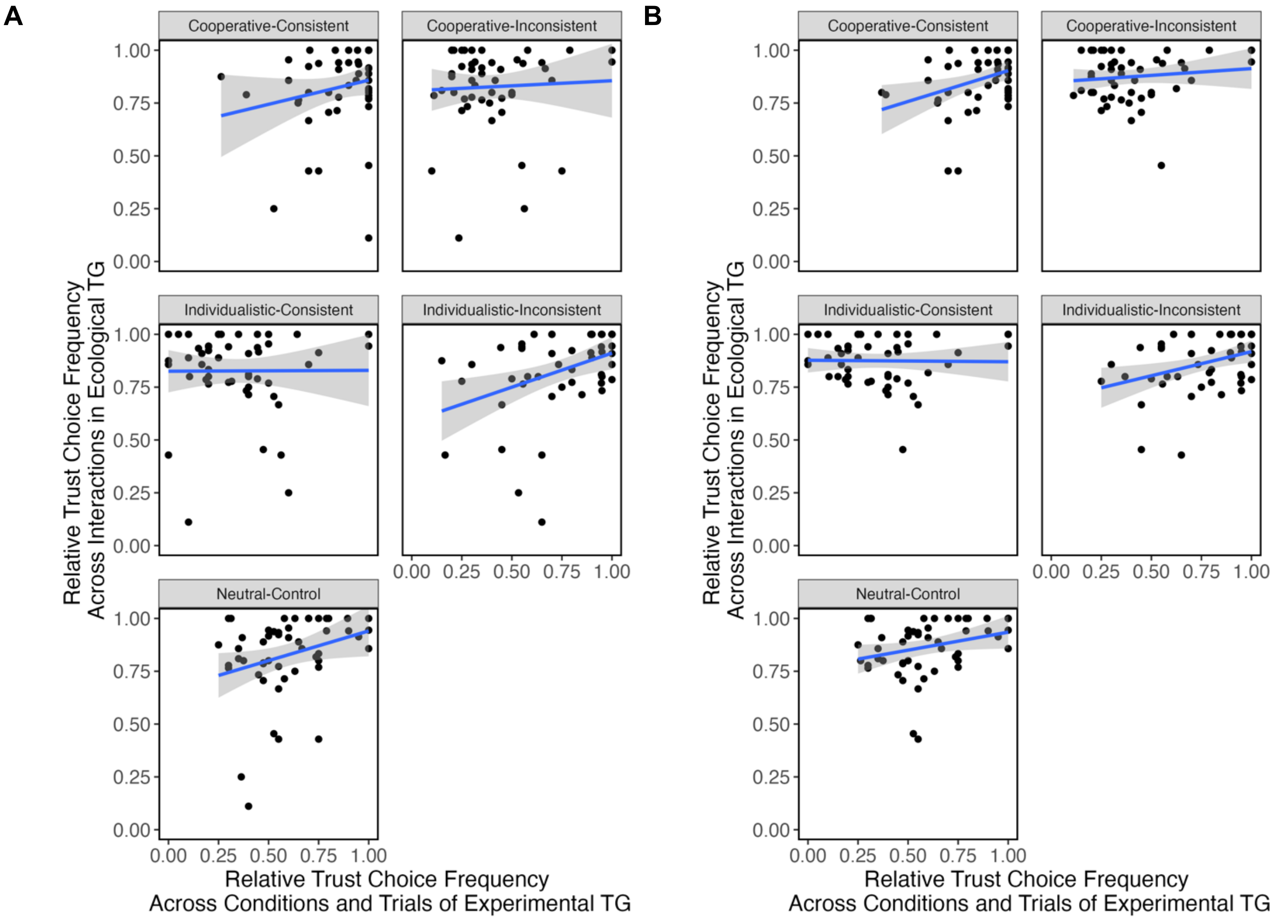

Note. Panel A: Before correction for influential data the blocks "Individualistic-Inconsistent" (F (1, 52) = 9.60, p = .003) and "Neutral-Control" (F (1, 52) = 4.39, p = .041) were significant at a p-level of 0.05. Panel B: After correction for influential data (Cook's Distance > 4/N), the blocks "Individualistic-Inconsistent" (F (1, 48) = 7.42, p = .009), and "Neutral-Control" (F (1, 49) = 4.07, p = .049) remained significant at a p-level of 0.05. Furthermore, the block "Cooperative-Consistent" (F (1, 48) = 7.12, p = .001) got significant at a p-level of 0.05.

Table S11. H5, H6: Regression Results Using Relative Trust Choice Frequency Across Interactions in Ecological Trust Game as Outcome and Electrocortical Potentials as Predictors

	b	SE (*b*)	*p*	CI LL	CI UL	*β*
(Intercept)	0.849	0.041	< 0.001	0.767	0.932	
Beta Power at FCz (mean across experiment)	0.019	0.034	0.577	-0.049	0.088	0.077
Mean Theta Power Difference at FCz (as bad – worse)	0.009	0.012	0.417	-0.014	0.033	0.112
Mean Theta Power Difference at F5, F6 (as good – better)	0.028	0.018	0.122	-0.008	0.064	0.216

Note. *Adjusted* R^2 = .005, SE (*b*) = standard error of the mean, CI = confidence interval, LL = lower level, UL = upper level, model specification: relative trust choice frequency across interactions in ecological trust game ~ mean beta power at FCz across trials + mean theta power difference at FCz (bad-as-excepted – worse-than-expected) + mean theta power difference at F5, F6 (good-as-expected – better-than-expected)

Table S12. H6: Regression Results Using Relative Trust Choice Frequency Across Interactions in Ecological Trust Game as Outcome and Electrocortical Potentials as Predictors

	b	SE (*b*)	*p*	CI LL	CI UL	*β*
(Intercept)	0.831	0.026	< 0.001	0.780	0.883	
Mean Theta Power Difference (as bad – worse)	0.010	0.012	0.409	-0.014	0.033	0.114
Mean Theta Power Difference (as good – better)	0.028	0.018	0.124	-0.008	0.063	0.213

Note. Adjusted R^2 = .018*, SE (b)* = standard error of the mean, CI = confidence interval, LL = lower level, UL = upper level, model specification: relative trust choice frequency across interactions in ecological trust game ~ mean theta power difference at FCz (bad-as-excepted – worse-than-expected) + mean theta power difference at F5, F6 (good-as-expected – better-than-expected)

Auxiliary Hypotheses

Table S13. Descriptives: Mean Response Time for Trust Game Choice by Block

Block	Mean Response Time	SD Response Time
Cooperative-Consistent	0.514	0.217
Cooperative-Inconsistent	0.511	0.228
Individualistic-Consistent	0.519	0.222
Individualistic-Inconsistent	0.519	0.232
Neutral-Control	0.512	0.23

Table S14. H7: Mixed Linear Regression Results Using Response Time for Trust Choice Execution in Laboratory Trust Game as the Outcome

	χ^2	df	*p*	*b*	SE (*b*)	CI LL	CI UL	*t*	*β*
(Intercept)	607.723	1	< 0.001	0.521	0.021	0.479	0.563	24.652	0.059
Feedback in $trial_{n-1}$	0.002	1	0.961	-0.001	0.012	-0.024	0.022	< 0.001	-0.003
**Theta Power at FCz**	**4.784**	**1**	**0.029**	**-0.003**	**0.002**	**-0.007**	**0.000**	**< 0.001**	**-0.080**
**Feedback in $trial_{n-1}$ X Theta Power at FCz**	**2.734**	**1**	**0.098**	**0.004**	**0.002**	**-0.001**	**0.008**	**1.653**	**0.090**

Note. Feedback in $trial_{n-1}$ (1 = worse than expected, 0 = bad as expected), bold values are significant at $p < 0.05$, *Adjusted* R^2 = .421, *CI* = 95% confidence interval, LL = lower level, UL = upper level, model specification: response time for trust choice in $trial_n$ ~ feedback in $trial_{n-1}$ * theta power at FCz + (1|subject)

Table S15. H8: Mixed Linear Regression Results Using Response Time for Trust Choice in Laboratory Trust Game as the Outcome

	χ^2	df	*p*	*b*	SE (*b*)	CI LL	CI UL	*t*	*β*
(Intercept)	758.516	1	< 0.001	0.521	0.019	0.484	0.559	27.541	0.049
**Feedback in trial_{n-1}**	**6.846**	**1**	**0.009**	**-0.015**	**0.006**	**-0.026**	**-0.004**	**< 0.001**	**-0.068**
Theta Power at F5, F6	0.256	1	0.613	0.000	< 0.001	-0.001	0.002	0.506	0.009
Feedback in trial_{n-1} X Theta Power at F5, F6	0.103	1	0.748	0.000	0.001	-0.002	0.003	0.321	0.008

Note. Feedback in trial_{n-1} (1 = good as expected, 0 = better than expected), bold values are significant at $p < 0.05$, *Adjusted* R^2 = .396, *CI* = 95% confidence interval, LL = lower level, UL = upper level, model specification: response time for trust choice in trial_n ~ feedback in trial_{n-1} * theta power at F5, F6 + (1|subject)

Table S16. H9: Mixed Linear Regression Results Using Feedback Related Negativity as the Outcome

	χ^2	df	*p*	*b*	SE (*b*)	CI LL	CI UL	*t*	*β*
(Intercept)	1.044	1	0.307	1.797	1.758	-1.647	5.241	1.022	0.032
Feedback in trial_n	1.643	1	0.200	-2.406	1.877	-6.083	1.273	< 0.001	-0.063
Trialnumber	0.035	1	0.853	-0.020	0.110	-0.236	0.195	< 0.001	-0.004
Feedback in trial_n X Trialnumber	0.164	1	0.686	0.063	0.155	-0.242	0.368	0.405	0.013

Note. Feedback in trial_n (1 = worse than expected, 0 = bad as expected), bold values are significant at $p < 0.05$, *Adjusted* R^2 = .102, *CI* = 95% confidence interval, LL = lower level, UL = upper level, model specification: feedback related negativity in trial_n ~ feedback in trial_n * trialnumber + (1|subject)

Table S17. H10: Mixed Linear Regression Results Using P2 as the Outcome

	χ^2	df	*p*	*b*	SE (*b*)	CI LL	CI UL	*t*	*β*
(Intercept)	0.030	1	0.862	-0.275	1.586	-3.379	2.829	< 0.001	0.016
Feedback in trial$_n$	0.707	1	0.400	1.780	2.116	-2.370	5.924	0.841	-0.035
Trialnumber	0.135	1	0.714	-0.045	0.124	-0.288	0.197	< 0.001	-0.012
Feedback in trial$_n$ X Trialnumber	1.956	1	0.162	-0.244	0.174	-0.586	0.098	< 0.001	-0.066

Note. Feedback in trial$_n$ (1 = good as expected, 0 = better than expected), bold values are significant at $p < 0.05$, *Adjusted* R^2 = .031, *CI* = 95% confidence interval, LL = lower level, UL = upper level, model specification: P2 at FCz in trial$_n$ ~ feedback in trial$_n$ * trialnumber + (1|subject)

References

Gosling, S. D., Rentfrow, P. J., & Swann, W. B. (2003). A very brief measure of the Big-Five personality domains. *Journal of Research in Personality*, *37*(6), 504–528. https://doi.org/10.1016/S0092-6566(03)00046-1

Hautzinger, M., & Bailer, M. (1993). *Allgemeine Depressions Skala (Kurzform)*. Beltz Test Gesellschaft.

Hautzinger, M., Bailer, M., Hofmeister, D., & Keller, F. (2012). *Allgemeine Depressionsskala: ADS*. Hogrefe.

Heimberg, R. G., Mueller, G. P., Holt, C. S., Hope, D. A., & Liebowitz, M. R. (1992). Assessment of anxiety in social interaction and being observed by others: The social interaction anxiety scale and the Social Phobia Scale. *Behavior Therapy*, *23*(1), 53–73. https://doi.org/10.1016/S0005-7894(05)80308-9

Jasielska, D., Rogoza, R., Zajenkowska, A., & Russa, M. B. (2021). General trust scale: Validation in cross-cultural settings. *Current Psychology*, *40*, 5019–5029.

Muck, P. M., Hell, B., & Gosling, S. D. (2007). Construct Validation of a Short Five-Factor Model Instrument. *European Journal of Psychological Assessment*, *23*(3), 166–175. https://doi.org/10.1027/1015-5759.23.3.166

Murphy, R. O., Ackermann, K. A., & Handgraaf, M. (2011). Measuring Social Value Orientation. *SSRN Electronic Journal*. https://doi.org/10.2139/ssrn.1804189

Rodrigues, J., Weiß, M., Mussel, P., & Hewig, J. (2022). On second thought … the influence of a second stage in the ultimatum game on decision behavior, electro-cortical correlates and their trait interrelation. *Psychophysiology*, *59*(7), e14023. https://doi.org/10.1111/psyp.14023

Schuermann, B., Endrass, T., & Kathmann, N. (2012). Neural correlates of feedback processing in decision-making under risk. *Frontiers in Human Neuroscience*, *6*. https://doi.org/10.3389/fnhum.2012.00204

Stangier, U., Heidenreich, T., Berardi, A., Golbs, U., & Hoyer, J. (1999). Die Erfassung sozialer Phobie durch Social Interaction Anxiety Scale (SIAS) und die Social Phobia Scale (SPS). [Assessment of social phobia by the Social Interaction Anxiety Scale (SIAS) and the Social Phobia Scale (SPS).]. *Zeitschrift Für Klinische Psychologie*, *28*(1), 28–36. https://doi.org/10.1026/0084-5345.28.1.28

Yamagishi, T., Akutsu, S., Cho, K., Inoue, Y., Li, Y., & Matsumoto, Y. (2015). Two-Component Model of General Trust: Predicting Behavioral Trust from Attitudinal Trust. *Social Cognition*, *33*(5), 436–458. https://doi.org/10.1521/soco.2015.33.5.436
